# Rehabilitation Interventions for Physical Capacity and Quality of Life in Adults With Post–COVID-19 Condition: A Systematic Review and Meta-Analysis

**DOI:** 10.1001/jamanetworkopen.2023.33838

**Published:** 2023-09-19

**Authors:** Dimitra V. Pouliopoulou, Joy C. Macdermid, Emily Saunders, Sue Peters, Laura Brunton, Erin Miller, Kieran L. Quinn, Tiago V. Pereira, Pavlos Bobos

**Affiliations:** 1 School of Physical Therapy, Faculty of Health Science, Western University, London, Ontario, Canada; 2 Roth McFarlane Hand and Upper Limb Centre, St Joseph’s Hospital, London, Ontario, Canada; 3 Department of Medicine, Temerty Faculty of Medicine, University of Toronto, Toronto, Ontario, Canada; 4 Health Technology Assessment Unit, Department of Pharmaceutical Sciences, Federal University of São Paulo, São Paulo, Brazil

## Abstract

**Importance:**

Current rehabilitation guidelines for patients with post–COVID-19 condition (PCC) are primarily based on expert opinions and observational data, and there is an urgent need for evidence-based rehabilitation interventions to support patients with PCC.

**Objective:**

To synthesize the findings of existing studies that report on physical capacity (including functional exercise capacity, muscle function, dyspnea, and respiratory function) and quality of life outcomes following rehabilitation interventions in patients with PCC.

**Data Sources:**

A systematic electronic search was performed from January 2020 until February 2023, in MEDLINE, Scopus, CINAHL, and the Clinical Trials Registry. Key terms that were used to identify potentially relevant studies included *long-covid*, *post-covid*, *sequelae*, *exercise therapy*, *rehabilitation*, *physical activity*, *physical therapy*, and *randomized controlled trial*.

**Study Selection:**

This study included randomized clinical trials that compared respiratory training and exercise-based rehabilitation interventions with either placebo, usual care, waiting list, or control in patients with PCC.

**Data Extraction and Synthesis:**

This study followed the Preferred Reporting Items for Systematic Reviews and Meta-Analyses. A pairwise bayesian random-effects meta-analysis was performed using vague prior distributions. Risk of bias was assessed using the Cochrane risk of bias tool version 2, and the certainty of evidence was evaluated using the GRADE system by 2 independent researchers.

**Main Outcomes and Measures:**

The primary outcome was functional exercise capacity, measured at the closest postintervention time point by the 6-minute walking test. Secondary outcomes were fatigue, lower limb muscle function, dyspnea, respiratory function, and quality of life. All outcomes were defined a priori. Continuous outcomes were reported as standardized mean differences (SMDs) with 95% credible intervals (CrIs) and binary outcomes were summarized as odds ratios with 95% CrIs. The between-trial heterogeneity was quantified using the between-study variance, τ^2^, and 95% CrIs.

**Results:**

Of 1834 identified records, 1193 were screened, and 14 trials (1244 patients; 45% female participants; median [IQR] age, 50 [47 to 56] years) were included in the analyses. Rehabilitation interventions were associated with improvements in functional exercise capacity (SMD, −0.56; 95% CrI, −0.87 to −0.22) with moderate certainty in 7 trials (389 participants). These improvements had a 99% posterior probability of superiority when compared with current standard care. The value of τ^2^ (0.04; 95% CrI, 0.00 to 0.60) indicated low statistical heterogeneity. However, there was significant uncertainty and imprecision regarding the probability of experiencing exercise-induced adverse events (odds ratio, 1.68; 95% CrI, 0.32 to 9.94).

**Conclusions and Relevance:**

The findings of this systematic review and meta-analysis suggest that rehabilitation interventions are associated with improvements in functional exercise capacity, dyspnea, and quality of life, with a high probability of improvement compared with the current standard care; the certainty of evidence was moderate for functional exercise capacity and quality of life and low for other outcomes. Given the uncertainty surrounding the safety outcomes, additional trials with enhanced monitoring of adverse events are necessary.

## Key Points

**Question:**

Are respiratory training and exercise-based rehabilitation interventions associated with improved functional exercise capacity in adults with post–COVID-19 condition?

**Findings:**

This systematic review, which incorporated a bayesian meta-analysis of 14 randomized clinical trials involving 1244 patients, found moderate-certainty evidence indicating that standardized rehabilitation interventions were associated with improvements in functional exercise capacity (standardized mean difference, −0.56; 95% credible interval −0.87 to −0.22) and had a 99% posterior probability of superiority compared with standard care. However, a high level of uncertainty and imprecision was observed concerning the probability of experiencing exercise-induced adverse events.

**Meaning:**

Although respiratory training and exercise-based rehabilitation interventions might be associated with improved functional exercise capacity in patients with post–COVID-19 condition, it is recommended that health care professionals closely monitor these patients during the implementation of such interventions to ensure patient safety until more definitive evidence is available.

## Teaser

This meta-analysis reviews studies investigating the association of rehabilitation interventions with physical capacity and quality of life in adults with post–COVID-19 condition (PCC).

## Introduction

The World Health Organization defines post–COVID-19 condition (PCC) as, “the continuation or development of new symptoms 3 months after the initial SARS-CoV-2 infection, with these symptoms lasting for at least 2 months with no other explanation.”^1^ Although COVID-19 was initially recognized as a respiratory illness, PCC symptoms can range from mild impairment to severe systemic disease, with many patients experiencing dozens of symptoms across multiple organ systems.^2,3,4^ At least 65 million individuals worldwide are estimated to have PCC, but the number is likely much higher due to many undocumented cases.^4^ The incidence is estimated at 10% to 30% of nonhospitalized cases, 50% to 70% of hospitalized cases, and 10% to 12% of vaccinated cases.^2^ In Canada, 14.8% of those who reported a previous positive test or a suspected infection of SARS-CoV-2 experienced symptoms at least 3 months after their infection,^5^ which translates into approximately 1.4 million Canadian adults or 4.6% of the Canadian population aged 18 years and older. Similarly, in the US, as of February 23, 2023, 14% of adults with a previous positive test for COVID-19 reported having experienced PCC symptoms at some point, and 6.5% reported current symptoms.^6^

The 5 most frequently observed symptoms include fatigue (58%), headache (44%), attention disorder (27%), hair loss (25%), and dyspnea (24%).^7^ Emerging evidence suggests that female sex, socioeconomic determinants of health (eg, lower income), inability to adequately rest in the early weeks after developing COVID-19, and comorbid conditions appear to be independent factors associated with increased risk for the development of PCC.^4,8^

According to a 2023 review,^2^ the existing research is insufficient to improve outcomes for people with PCC. There is an urgent need for evidence-based rehabilitation interventions to support people affected by PCC^9,10,11,12^ because current guidelines are primarily based on expert opinion and observational data.^13,14^ The latest systematic review^15^ with meta-analysis on rehabilitation interventions for patients with PCC included a total of 3 trials^16,17,18^ (233 patients with PCC) and suggested that rehabilitation interventions may be associated with improvements in functional exercise capacity, but the association of rehabilitation interventions with respiratory function was inconsistent across studies. The accuracy of these early-evidence syntheses is likely to be compromised due to the small number of included studies. Since then, 11 additional trials^19,20,21,22,23,24,25,26,27,28,29,30^ have emerged. The increased attention on PCC now allows us to conduct a more comprehensive, methodologically sound, and stable analysis. The purpose of this meta-analysis is to assess whether rehabilitation interventions are associated with improvements in physical capacity (functional exercise capacity, muscle function, dyspnea, and respiratory function) and quality of life in adults with PCC.

## Methods

Our protocol was registered in PROSPERO database and is available in the eAppendix in Supplement 1. We followed the Preferred Reporting Items for Systematic Reviews and Meta-Analyses (PRISMA) reporting guideline to report this systematic review.^31^

### Search Strategy and Information Sources

After consulting with a librarian, a systematic electronic search of the literature was performed from January 2020 until February 2023, in MEDLINE (via Ovid), Scopus, CINAHL, and the Clinical Trials Registry. Examples of the keywords that were used to identify potentially relevant studies were *long-covid*, *post-covid, sequelae*, *exercise therapy*, *rehabilitation*, *physical activity*, *physical therapy*, and *randomized controlled trials.* The full research strategy is summarized in eTable 1 in Supplement 1. Additionally, we conducted a manual search of the reference lists of the included studies to identify any additional studies not retrieved in the electronic search.

### Study Selection and Data Extraction

We included randomized clinical trials that compared rehabilitation interventions such as respiratory training aerobic exercises and resistance exercises with either placebo, usual care, waitlist, or control in adults with PCC.^1^ We did not pose any restrictions with regard to comorbidities or medication use concurrently with the rehabilitation protocol. Trials that used medication-only treatments without a rehabilitation component and nonrandomized studies were excluded.

As part of the study selection, 2 independent researchers (D.V.P. and E.S.) screened titles and abstracts of articles, reviewed the articles that met inclusion criteria in initial screening at the full text level, and extracted data from the eligible studies. We extracted authors, year of publication, participant and intervention characteristics, and outcome data.

### Outcomes

The primary outcome was functional exercise capacity, measured with the 6-minute walking test. Our secondary outcomes were fatigue; functional leg strength and endurance as measured by the 30-second sit-to-stand test; dyspnea; respiratory function; and quality of life. The time point of the primary and secondary outcomes was the shortest time point available upon completion of the rehabilitation program. Respiratory function was assessed through forced expiratory volume in the first second (FEV_1_) and forced vital capacity (FVC). If a study used more than 1 measure to assess dyspnea and quality of life, we planned to extract the measure reported as the primary outcome. We also assessed the safety of the rehabilitation interventions by extracting any treatment-emergent adverse events (including serious adverse events) across all studies. Safety outcomes were assessed at the last follow-up available.

### Risk of Bias Assessment and Certainty of the Evidence

To assess the risk of bias, 2 independent reviewers (D.V.P. and E.S.) used the Cochrane risk of bias tool version 2.^32^ We assessed selection bias, performance bias, detection bias, attrition bias, and reporting bias. We used the GRADE approach^33,34,35,36,37,38,39^ to evaluate the certainty of evidence with regard to risk of bias, inconsistency, indirectness, imprecision, and publication bias. Discrepancies were resolved with a senior research team member (P.B.).

### Statistical Analysis

We described variables with a normal distribution as mean (SD) and those with a skewed distribution as median (IQR). Categorical variables were presented as numbers (percentages).

The different rehabilitation interventions were compared against a common comparison group, termed *control* (ie, placebo, sham, waiting list, or usual care). We used bayesian pairwise, random-effects, meta-analysis models to summarize results across trials. We chose random-effects models to account for the anticipated clinical and methodological diversity between studies.^40^

For continuous outcomes, we used the normal likelihood and an identity link. For binary outcomes, we used the binomial likelihood and the logit link. All models were fitted with noninformative prior distributions. We used normal prior distributions with a mean of 0 and a large variance for treatment effects. For the main analysis, we used uniform, vague prior distributions for the between-trial variance. In sensitivity analyses, we used empirical prior distributions for the between-trial variance. We quantified the between-trial heterogeneity using the between-study variance, τ^2^, and 95% credible intervals (95% CrIs). In the presence of substantial heterogeneity, we performed sensitivity analysis on the basis of allocation concealment that was defined a priori as potential moderator.

For continuous outcomes, summary results were presented as standardized mean differences (SMDs) with 95% CrIs. We coined all outcomes such that negative treatment effects (SMD <0) indicate better outcomes in the intervention group compared with the comparison. We calculated the probability of rehabilitation interventions being superior to usual care (ie, probability of the true SMD <0) and the probability of rehabilitation interventions providing a treatment effect more pronounced than the between-group minimum important difference (MID) of 0.30 SD units (ie, the probability that the true SMD is ≤−0.30).

For binary outcomes, summary results were presented as odds ratios (ORs) with 95% CrIs, with an OR greater than 1 representing a higher risk of the event among patients who received the intervention than those who received the comparison.

Parameters were estimated via Markov chain Monte Carlo methods. We used a burn-in period of 50 000 iterations and 3 chains with 100 000 simulations each (300 000 in total). Summary estimates were obtained via posterior medians (the 2.5 percentile and the 97.5 percentile). We monitored model convergence via the Brooks-Gelman-Rubin *R* statistic and trace plots. Model diagnostics also included visual inspection of autocorrelation plots and the posterior densities.

We used the Egger test for continuous outcomes and Peters test for binary outcomes to assess for publication bias. In the presence of large between-study heterogeneity, we used allocation concealment as a moderator. A 2-sided *P* < .10 was considered evidence of publication bias.^41^ All analyses were conducted in OpenBUGS^42^ statistical software version 3.2.3 (University of Cambridge) and Stata statistical software version 16 (StataCorp). Details on the prior distributions and models are provided in the eMethods in Supplement 1.

## Results

**Figure 1.**
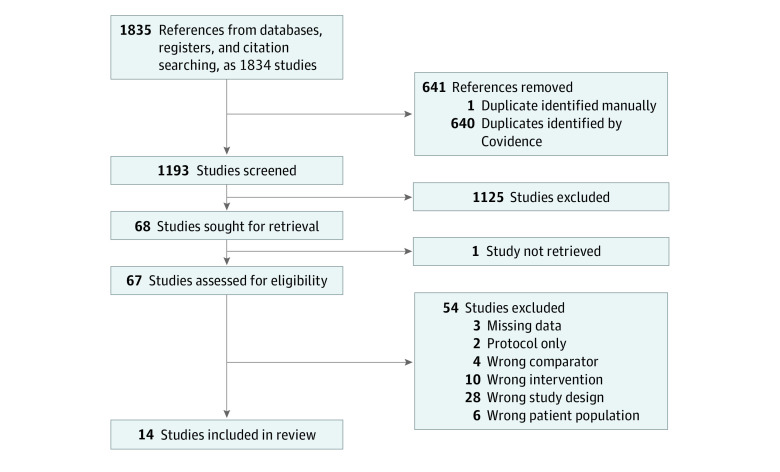
Flowchart Showing Study Selection

Our search identified 1834 records. After removal of duplicates, we carried out title and abstract screening of 1193 references, leaving 67 articles selected for full text review. Of these studies, 14 trials (15 records) were deemed eligible (Figure 1). Excluded references from the full text screening and the reasons for exclusion are provided in eTable 2 in Supplement 1. The characteristics of the included studies are summarized in Table 1.

**Table 1.** Characteristics of the Included Studies

Source	Primary outcome	Population	Age, mean (SD) y	Participants, No.	Women, No. (%)	Men, No. (%)	Intervention	Control	Length of inpatient stay, d	Level of dyspnea
Teixeira DO Amaral et al,^18^ 2022	6MWT, FEV_1_^a^ and FVC^a^	Hospitalized patients due to COVID-19 (6.2% ICU patients)	52.0 (10.8)	32	17 (53.1)	15 (46.9)	Clinician supported, home-based aerobic and resistance exercise program	Usual care	6.8	NI
Capin et al,^20^ 2022	30-s STS, and dyspnea^b^	Hospitalized patients due to COVID-19 (with and without ICU stay)	53.0 (10)	41	18 (44.0)	23 (56.0)	Supervised breathing and clearance techniques, high-intensity strength training, aerobic and cardiovascular exercise, balance exercises, functional activities, stretching, coaching, and motivational interviewing	Usual care	6.0	Moderate to severe
Del Corral et al,^21^ 2023	30-s STS, health-related quality of life,^c^ FEV_1_,^a^ and FVC^a^	Patients reporting persistent fatigue and dyspnea following PCR diagnosis of COVID-19 infection (32.0% hospital admission rate; 6.0% ICU patients)	46.5 (10.2)	88	14 (16.0)	74 (84.0)	Supervised inspiratory muscle training and respiratory muscle training	Sham	NI	NI
De Souza et al,^19^ 2021	30-s STS	Nonhospitalized patients	NI	196	NI	NI	Supervised low-intensity pulmonary rehabilitation	Usual care	NA	NI
Jimeno-Almazán et al,^22^ 2023 and Jimeno-Almazán et al,^23^ 2022	Dyspnea,^b^ quality of life,^d^ FEV_1_,^a^ and FVC^a^	Nonhospitalized patients with a PCR diagnosis of COVID-19 presenting with persistent symptoms	45.3 (9.7)	39	29 (74.3)	10 (25.7)	Supervised resistance training combined with aerobic training	Usual care	NA	Mild and moderate
Li et al,^16^ 2022	6MWT, quality of life,^d^ FEV_1_,^a^ and FVC^a^	Patients reporting persistent moderate dyspnea (mMRC >2-3) after inpatient treatment for COVID-19	50.6 (11.0)	119	66 (55.5)	53 (44.5)	Unsupervised breathing control and thoracic expansion, aerobic exercise, and lower limb muscle strengthening exercises	Usual care	26.2	Moderate
Liu et al,^17^ 2020	6MWT	Hospitalized patients due to COVID-19 aged ≥65 y	67.5 (7.8)	72	23 (32.0)	49 (68.0)	Supervised respiratory muscle training, cough exercise, diaphragmatic training, stretching exercise, and unsupervised home exercise	Usual care	NI	NI
McNarry et al,^24^ 2022	Dyspnea^e^ and quality of life^f^	Patients reporting persistent dyspnea following COVID-19 infection	46.6 (12.2)	281	247 (88.0)	34 (12.0)	Unsupervised inspiratory muscle training	Usual care	NI	Moderate
Okan et al,^25^ 2022	6MWT, dyspnea,^b^ quality of life,^g^ FEV_1_,^a^ and FVC^a^	Patients presenting with persistent dyspnea following COVID-19–induced pneumonia	50.0 (12.8)	52	25 (48.0)	27 (52.0)	Supervised breathing exercise	Usual care	9.5	Moderate
Phillip et al,^26^ 2022	Dyspnea^h^ and quality of life^i^	Patients reporting persistent dyspnea following COVID-19 infection (17.0% hospital admission rate)	49.5 (12.0)	150	26 (17.5)	124 (82.5)	Supervised posture and breathing exercises	Usual care	NI	Mild
Rodriguez-Blanco et al,^30^ 2023	6MWT, 30-s STS, dyspnea^h^	Patients with symptoms attributed to COVID-19 by medical services	40.7 (13.4)	48	26 (54.2)	22 (45.8)	Breathing and strength-based exercises	Usual care	NI	Mild
Romanet et al,^27^ 2022	Dyspnea^b^ and quality of life^d^	Patients reporting persistent dyspnea (mMRC >1) following COVID-19–related acute respiratory distress syndrome (ICU admission)	58.2 (12.5)	60	23 (38.1)	37 (61.9)	Supervised endurance training rehabilitation	Usual care	26.0	Moderate
Sari et al,^28^ 2023	6MWT	PCC with pulmonary involvement (83.0% hospitalized, 17.0% ICU)	56.2 (4.5)	24	8 (33.3)	16 (66.7)	Nonsupervised breathing exercises, resistance exercises, and inspiratory muscle training	Usual care	NI	Mild and moderate
Şahın et al,^29^ 2023	6MWT, dyspnea,^b^ FEV_1_,^a^ FVC,^a^ and quality of life^g^	Patients hospitalized with PCC (ICU and ward for >10 d)	60.7 (8.2)	42	14 (33.3)	28 (66.7)	Clinician supported breathing exercises, strength exercises, and walking program	Usual care	11.5	Moderate

Abbreviations: 6MWT, 6-minute walking-test; FEV_1_, forced expiratory volume in the first second; FVC, forced vital capacity; ICU, intensive care unit; mMRC, modified Medical Research Council Dyspnea Scale; NA, not applicable; NI, no information; PCC, post–COVID-19 condition; PCR, polymerase chain reaction; STS, sit-to-stand test.

^a^ Calculated as estimated percentages.

^b^ Measured with the mMRC.

^c^ Measured with the Euroqol-5 Dimension, 5-Level (EQ-5D-5L) Questionnaire.

^d^ Measured with the 12-Item Short Form Survey (SF-12) mental component score and physical component score.

^e^ Measured with the Transition Dyspnea Index.

^f^ Measured with The King’s Brief Interstitial Lung Disease Questionnaire.

^g^ Measured with the St George Respiratory Questionnaire.

^h^ Measured with the Dyspnea-12 Questionnaire.

^i^ Measured with the physical health component and mental health component of the 36-Item Short Form Survey (SF-36).

### Description of the Population

The analysis included 1244 participants (median [IQR] age, 50 [47-56] years; 45% female participants). Six trials^16,17,18,20,27,29^ included patients who had previously been hospitalized due to a COVID-19 infection (range of intensive care unit admission, 6.2%-100%), and 3 trials^19,22,23,30^ (4 records) included patients who had not been hospitalized following SARS-CoV-2 infection. Five trials^21,24,25,26,28^ included a mixed population of both patients who had been hospitalized following initial COVID-19 infection and those who had not been hospitalized following initial COVID-19 infection. The level of dyspnea of the participants at the baseline ranged from mild to severe.

### Description of the Interventions

The most common interventions in the treatment group were breathing exercises, either alone^19,21,24,25,26,30^ (6 trials [815 participants]), or in combination with resistance and/or aerobic training^16,17,20,28,29^ (5 trials [298 participants]). In 2 trials^18,22,23^ (3 records [71 participants]), the intervention included strengthening and aerobic exercises without a breathing exercise component, and 1 trial^27^ (60 participants) included only aerobic exercises. According to the TiDiER checklist,^43^ 12 trials (86%) gave appropriate descriptions of the intervention components, 11 trials (79%) stated the dose and duration of the intervention, and 9 trials (64%) described the tailoring process. A detailed analysis of the reporting of each component for each study is summarized in eTable 3 in Supplement 1. The most common comparator was usual care in the form of respiratory training and exercise-based, self-management education^16,17,18,19,20,22,23,24,25,26,27,28,29,30^ (13 studies [14 records; 1156 participants]). A single study^21^ (88 participants) used sham or placebo comparisons in the form of a respiratory training with no resistance. Minimum exercise dose was not well defined for our population. The most common limitations of unsupervised respiratory training (ie, usual care) were wrong use of equipment and ineffective execution of the breathing techniques. Hence, all comparators will be referred to as *usual care*.

### Risk of Bias

**Figure 2.**
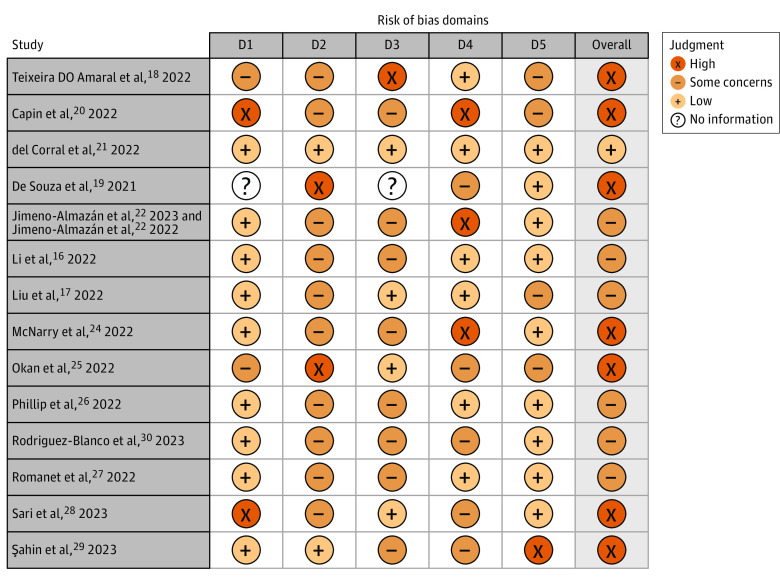
Risk of Bias Assessment Domains include bias arising from the randomization process (D1), bias due to deviations from intended intervention (D2), bias due to missing outcome data (D3), bias in measurement of the outcome (D4), and bias in selection of the reported result (D5).

Figure 2 summarizes the risk of bias judgment for each individual trial. Overall risk of bias was deemed low in 1 study^21^ (7%), deemed as having some concerns in 6 trials^16,17,22,23,26,27,30^ (7 records [43%]), and deemed high in 7 trials^18,19,20,24,25,28,29^ (50%). Risk of bias arising from the randomization process and the selection of the reported outcomes was deemed low in 9 trials^16,19,21,22,23,24,26,27,29,30^ (10 records [64%]). Bias due to deviations of the included intervention was deemed low in 2 trials^21,28^ (14%). The most common sources of bias due to deviations of the included intervention were nonblinding of the patients and/or the clinicians. Bias due to missing outcome data was deemed low in 4 trials^17,21,25,29^ (29%). Bias arising from the measurement of the outcome was deemed low in 6 trials^16,17,18,21,26,27^ (43%).

### Primary Outcome: Functional Aerobic Capacity

**Figure 3.**
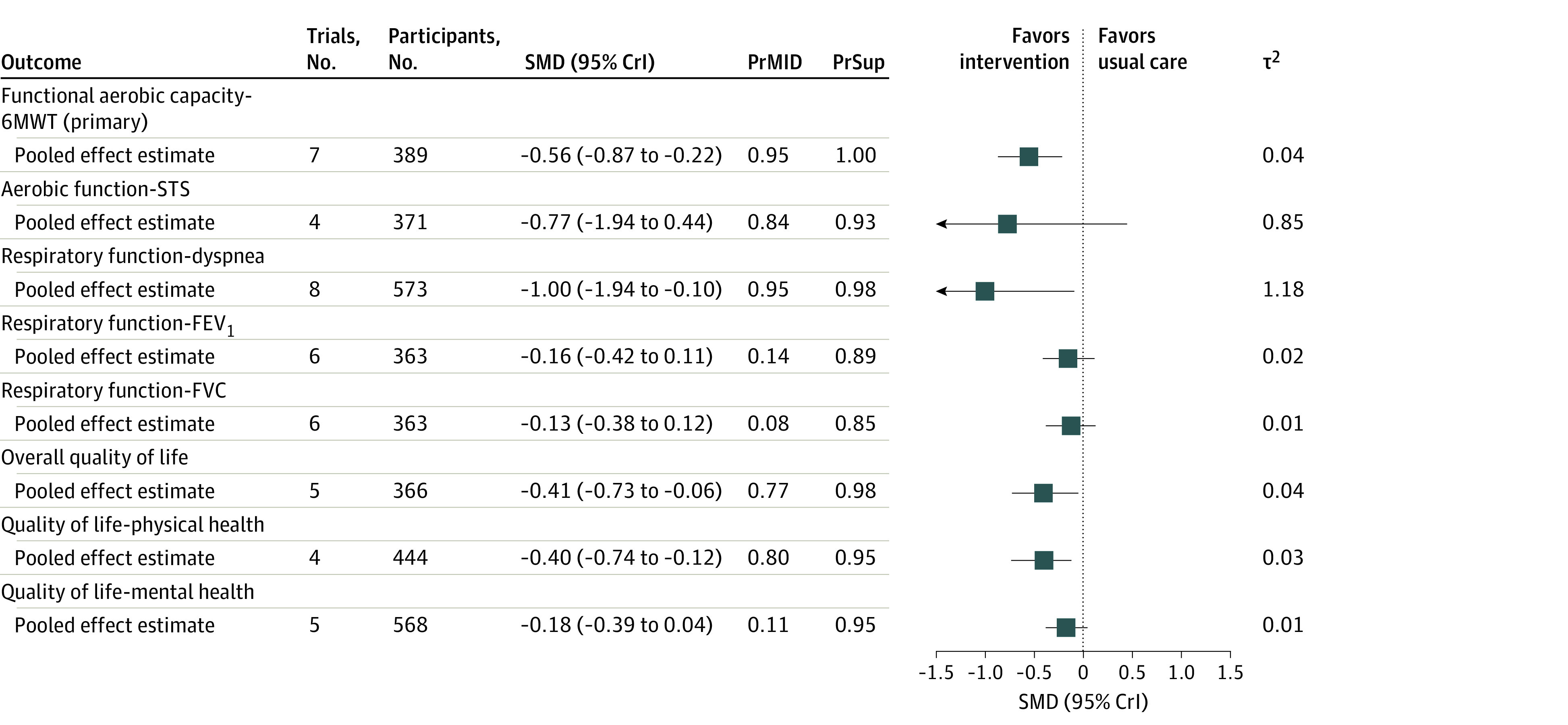
Treatment Outcomes of Rehabilitation Interventions vs Usual Care All results are based on a bayesian random-effects model. Results are reported as standardized mean differences (SMDs) and 95% credible intervals (CrIs). PrSup is the probability of the superiority of rehabilitation interventions over usual care (ie, the probability that the SMD is <0). PrMID is the probability of the true treatment effect being equal to or more exacerbated than the between-group minimal important difference (MID) threshold (ie, the probability that the SMD is ≤−0.30). 6MWT indicates 6-minute walking test; FEV_1_, forced expiratory volume in the first second; FVC, forced vital capacity; STS, 30-second sit-to-stand test.

A total of 7 trials^16,17,18,25,28,29,30^ involving 389 participants with PCC reported treatment outcomes on functional exercise capacity measured by the 6-minute walking test. The median (IQR) follow-up time for evaluating the primary outcome after randomization was 6 (5.5-7.0) weeks. Rehabilitation interventions were associated with a greater improvement in functional exercise capacity compared with usual care (SMD, −0.56; 95% CrI, −0.87 to −0.22) (Figure 3 and eFigure 1 in Supplement 1). Rehabilitation interventions demonstrated a 99.6% posterior probability of superiority and were associated with 95% probability of reaching the MID threshold when compared with controls. Specifically, patients in the intervention group were able to cover a mean (SD) of 35.84 (6.55) m more than patients in the usual care group during the 6-minute walking test (95% CI, 34.97 m to 36.71 m). The value of τ^2^ (0.04; 95% CrI, 0.00 to 0.60) indicated low heterogeneity for exercise capacity. The confidence in the certainty of evidence was moderate and was rated down due to high risk of bias (Table 2).

**Table 2.** Grade Rating of Studies

Outcomes	Randomized clinical trials, No.	Participants, No.	Effect size estimate, (95% CrI)	Certainty of evidence (GRADE)^a^	Reason for downgrade
Functional exercise capacity (6MWT)	7	389	−0.56 (−0.87 to −0.22)^b^	Moderate	Downgraded for high risk of bias
Functional leg strength and endurance (STS)	4	371	−0.77 (−1.94 to 0.44)^b^	Very low	Downgraded for high risk of bias and imprecision
Dyspnea	8	573	−1.00 (−1.94 to −0.10)^b^	Low	Downgraded for high risk of bias and inconsistency
Respiratory function (FEV_1_, %)	6	363	−0.16 (−0.42 to 0.11)^b^	Low	Downgraded for high risk of bias and indirectness
Respiratory function (FVC, %)	6	363	−0.13 (−0.38 to 0.12)^b^	Low	Downgraded for high risk of bias and indirectness
Quality of life (overall)	5	366	−0.41 (−0.73 to −0.06)^b^	Moderate	Downgraded for high risk of bias
Quality of life (mental health)	5	568	−0.18 (−0.39 to 0.04)^b^	Moderate	Downgraded for high risk of bias
Quality of life (physical health)	4	444	−0.40 (−0.74 to −0.12)^b^	Moderate	Downgraded for high risk of bias
Adverse events	5	544	1.68 (0.32 to 9.94)^c^	Low	Downgraded for high risk of bias

Abbreviations: 6MWT, 6-minute-walking-test; CrI, credible interval; FEV_1_, forced expiratory volume in the first second; FVC, forced vital capacity; GRADE, grading of recommendations, assessment, development, and evaluations; STS, sit-to-stand test.

^a^ The certainty of evidence was rated using the GRADE system, with a rating of high indicating we are very confident that the true effect lies close to the effect size estimate; moderate indicating we are moderately confident in the effect size estimate and that the true effect is likely to be close to the estimate of effect size, but there is a possibility that it is substantially different; low indicating our confidence in the effect size estimate is limited and that the true effect may be substantially different from the size estimate of effect; and very low indicating we have very little confidence in the effect size estimate and that the true effect is likely to be substantially different from the size estimate of effect.

^b^ Reported as a standardized mean difference.

^c^ Reported as an odds ratio.

### Fatigue

The available evidence on fatigue outcomes was limited and could not be synthesized. A single study^21^ measured the presence or absence of fatigue during everyday activities as a binary outcome without quantifying it. A total of 2 studies^28,29^ assessed only exercise-induced fatigue levels, and 3 studies (4 records)^22,23,24,30^ did not adequately report the circumstances under which fatigue was measured. Additionally, 2 studies^20,26^ recorded the presence of exercise-induced fatigue as an adverse event.

### Functional Lower Limb Muscle Strength and Endurance

Four trials^19,20,21,30^ reported treatment outcomes on lower limb muscle function among 371 participants with PCC. The summary point estimate suggested that rehabilitation interventions could be associated with improvements in lower limb muscle function when compared with usual care. However, given that the CrI included both positive and negative values, the magnitude and direction of the association remain uncertain (SMD, −0.77; 95% CrI, −1.94 to 0.44) (Figure 3and eFigure 2 in Supplement 1). Rehabilitation interventions demonstrated a 93% posterior probability of superiority and were associated with 84% probability of reaching the MID threshold when compared with usual care. The value of τ^2^ (0.8; 95% CrI, 0.15 to 8.70) indicated substantial statistical heterogeneity for the 30-second sit to stand test. The confidence in the certainty of evidence of the treatment outcomes was very low and was rated down due to high risk of bias, imprecision, and indirectness (Table 2).

### Dyspnea

Eight trials (9 records)^20,22,23,24,25,26,27,29,30^ involving 573 participants with PCC reported treatment outcomes on dyspnea. Rehabilitation interventions were associated with a greater improvement in functional exercise capacity compared with usual care (SMD, −1.00; 95% CrI, −1.94 to −0.10) (Figure 3 and eFigure 3 in Supplement 1). Rehabilitation interventions were associated with a 98% posterior probability of superiority when compared with usual care and were associated with a 95% probability to reach the MID threshold. The magnitude of τ^2^ indicated substantial statistical heterogeneity (τ^2^, 1.18; 95% CrI, 0.36 to 5.50). Heterogeneity was partially explained when adjusting for allocation concealment (eFigure 4 in Supplement 1). The confidence in the certainty of evidence was low and rated down due to high risk of bias and inconsistency (Table 2).

### Respiratory Function: FEV_1_ and FVC

A total of 6 trials (7 records)^16,18,21,22,23,25,29^ reported treatment outcomes on FEV_1_ (estimated percentage) and FVC (estimated percentage) among 363 participants with PCC. We found no difference between rehabilitation interventions and usual care in either FEV_1_ (SMD, −0.16; 95% CrI, −0.42 to 0.11) or FVC (SMD, −0.13; 95% CrI, −0.38 to 0.12) (Figure 3 and eFigure 5 and eFigure 6 in Supplement 1). Rehabilitation interventions demonstrated 89% and 85% posterior probability of superiority when compared with usual care for FEV_1 _and FVC respectively. The magnitude of τ^2^ indicated very low statistical heterogeneity for both FEV_1_ (τ^2^, 0.02; 95% CrI, 0.00 to 0.28) and FVC (τ^2^, 0.01; 95% CrI, 0.00 to 0.23). The confidence in the certainty of evidence was low for both outcomes and downgraded for high risk of bias and indirectness (Table 2).

### Quality of Life

Five trials^21,24,25,27,29^ reported treatment outcomes on overall quality of life among 366 participants with PCC. Rehabilitation interventions were associated with larger improvement in quality of life compared with the comparison group (SMD, −0.41; 95% CrI, −0.73 to −0.06) (Figure 3 and eFigure 7 in Supplement 1). Rehabilitation interventions were associated with a 98.4% posterior probability of being superior to the comparison group and were associated with a 76.9% probability to reach the MID threshold. The magnitude of τ^2^ indicated very low statistical heterogeneity (τ^2^, 0.04; 95% CrI, 0.00 to 0.63). The confidence in the certainty of evidence was moderate and rated down for high risk of bias (Table 2). Figure 3 and eFigure 8 and eFigure 9 in Supplement 1 display the association of rehabilitation interventions with physical and mental health separately.

### Adverse Events

Five trials^17,19,27,28,29^ did not provide any information on adverse events, and 4 trials (5 records)^20,22,23,25,30^ reported no adverse events related to the intervention. Adverse events were reported in 5 trials.^16,20,21,24,26^ However, we did not find compelling evidence for a difference in the odds of adverse events occurring. The wide 95% CrI indicates a high level of uncertainty and imprecision in this estimate (OR, 1.68; 95% CrI, 0.32 to 9.94) (eTable 4 and eTable 5 in Supplement 1).

### Publication Bias, Autocorrelation, and Model Convergence

We found no indication of publication bias (eTable 6 in Supplement 1). No major concerns of autocorrelation or nonconvergence were identified. The autocorrelation and the trace plots for the main outcome are presented in eFigure 10 and eFigure 11 in Supplement 1.

### Sensitivity Analysis

Analyses considering empirical prior distribution for the between-trial variability rendered virtually identical results (eTable 7 in Supplement 1). Analyses incorporating a thinning of 10 are presented in eFigure 12 and eFigure 13 in Supplement 1.

## Discussion

Our meta-analysis of 14 randomized clinical trials examining different rehabilitation programs for people with PCC found that the patients undergoing rehabilitation experienced larger improvement in functional exercise capacity, dyspnea, and quality of life outcomes than patients receiving usual care. The analysis consistently showed that rehabilitation interventions had a greater probability of being superior to usual care across all outcomes, with probabilities ranging between 85% and 99%. Additionally, rehabilitation interventions were associated with higher probability of reaching the predefined between-group MID threshold for functional aerobic capacity, functional lower limb strength and endurance, dyspnea, and quality of life, with probabilities ranging between 84% and 95%.

### Clinical Implications

Substantial advances in vaccines and prevention strategies of acute SARS-CoV-2 infection may help reduce the burden of PCC. However, as pharmacological advances improve the prognosis of people living with comorbidities who develop an acute SARS-CoV-2 infection, the number of patients living with PCC is expected to grow. Thus, it is of high importance to develop a safe and effective strategy that will be based on high-quality evidence and will be applicable to a broad population. Given the small number of randomized clinical trials and the recent emergence of PCC, it is of no surprise that both the clinical practice and the evidence is rapidly evolving. Current treatment guidelines based on expert opinion suggest a supervised, individualized, symptom-based approach with close monitoring for adverse events such as orthostatic intolerance and postexertional symptom exacerbation.^2,55^ Yet, current standard care is still provided in the form of self-management recommendations in an ambulatory or home setting. The results of this study indicate that respiratory training and exercise-based rehabilitation interventions may have a greater benefit than current standard care. Inspiratory muscle training requires strict control and high adherence to achieve effective results.^56,57^ Research on people with chronic obstructive pulmonary disease has shown that education-based, nonsupervised rehabilitation programs are likely to be less effective than clinician-guided ones, mainly due to the wrong use of equipment, ineffective execution of the breathing techniques, and lower adherence.^56,57^ Additionally, we found a high level of uncertainty and imprecision in the probability of experiencing adverse events. These results further highlight the importance of supervised interventions with continuous monitoring and tailoring to ensure fidelity and patient safety until indicated otherwise.

Female sex and inability to adequately rest in the early weeks after developing COVID-19 are associated with increased risk of development of PCC.^4^ Because women often take on societal roles such as caring and household duties, which may not allow for adequate rest in the early weeks after infection, women may face increased risk for PCC due to both biological and social factors. Yet, only 45% of the population in the included studies were women. This finding highlights an important research gap that should be addressed in future studies.

Despite research indicating that fatigue is the most frequently observed symptom among individuals with PCC, we found limited evidence quantifying the association of rehabilitation interventions with fatigue during everyday activities. This scarcity of high-quality evidence for managing fatigue has been previously underscored by de Sire et al^58^ in a systematic review that explored the association of rehabilitation with fatigue in patients with PCC. The review^58^ identified only 1 observational study and 5 intervention-based studies that lacked a control group. Evidence-based practice hinges on high-quality research, and this considerable gap in the current evidence should be addressed in future studies.

### Strengths and Limitations

Although several systematic reviews on PCC have emerged,^38,39,40,41,42,43,44,45,46,47,48,49,50^ our findings add to the literature on PCC recovery in several ways. Some of the limitations of the currently published evidence are the lack of a meta-analysis component^44,45,46,47,48,49,50,51,52,53^ and the synthesis of data from both acute care patients and PCC patients in the same analysis.^54^ This study is the first, to our knowledge, to conduct a systematic review with a bayesian meta-analysis on rehabilitation interventions for patients with PCC. Our findings are in agreement with a previous systematic review with meta-analysis on PCC. Chen et al^15^ included a total of 3 trials (233 patients with PCC) and found that rehabilitation interventions were associated with a clinical important difference in favor of pulmonary rehabilitation for exercise capacity, but the outcomes on respiratory function were inconsistent across studies. Our review identified 11 additional trials (ie, a total of 14 trials with 1244 participants) and examined a full range of outcomes. To increase the robustness of our results we calculated the probability to indicate superiority of the rehabilitation interventions when compared with usual care, and we prespecified all of our analyses. The robustness and accuracy of our results are further supported by the low between-trial heterogeneity and absence of publication bias. In addition, we used an extended and comprehensive search strategy and searched all relevant sources to retrieve all the potentially eligible randomized clinical trials. We therefore believe that it is unlikely that we missed any relevant trials.

The quality of our analysis is limited by the quality of the underlying data. Although we only included randomized clinical trials, these studies were not without flaws, and the overall evidence grade was deemed to be moderate. Bias in the included studies in terms of allocation concealment, blinding, and missing data could have led to an overestimation of the treatment outcomes. Currently, there is no criterion standard measurement tool for functional aerobic capacity, fatigue, dyspnea, and quality of life in patients with PCC. Therefore, it is possible that certain outcomes did improve, but the measurement tools that were chosen by the researchers did not have the ability to measure this change, which could have led to an underestimation of the treatment outcomes. Some trials included estimates from a per-protocol analysis only, which may have also led to overestimation of the treatment outcomes. Even though female sex is associated with higher risk of PCC, only 45% of the participants in the included studies were women, which could have potentially reduced the external validity of our results. We did not pose any restriction on concurrent medication use with the rehabilitation protocol. Including studies that allowed for concurrent medications increased the chances of overestimating the treatment outcomes due to potential synergy effects. However, not allowing for concurrent medications would have made our results inapplicable to a large proportion of people living with comorbidities who depend on several medication-based treatments. Because comorbidities are associated with higher risk of development of PCC, we believe that not allowing for concurrent medication would have been unfeasible, impractical, and unethical. Our analysis was focused on the shortest time point available after the completion of the interventions, which allowed us to measure the effects of the interventions more accurately and minimize any potential impact from external trial factors that could influence treatment outcomes but prevented us from assessing any associations in the long-term follow-ups.

## Conclusions

Rehabilitation interventions demonstrated an association with improved outcomes in functional exercise capacity, dyspnea, and quality of life, with a high probability of improvement compared with the current standard of care. The certainty of evidence was moderate for functional exercise capacity and quality of life and low for other outcomes. Given the uncertainty surrounding our safety outcomes, additional trials with enhanced monitoring of adverse events are necessary.

## Notes

Supplement 1. **eAppendix.** Study Protocol **eTable 1.** Research Strategy **eMethods.** Models **eTable 2.** Excluded Studies–Reasons for Exclusion **eTable 3.** Tidier **eFigure 1.** Caterpillar Plot for Exercise Capacity **eFigure 2.** Caterpillar Plot for Lower Limb Muscle Function **eFigure 3.** Caterpillar Plot for Dyspnea **eFigure 4.** Sensitivity Analysis for Dyspnea (Allocation Concealment) **eFigure 5.** Caterpillar Plot for Respiratory Function (FEV1) **eFigure 6.** Caterpillar Plot for Respiratory Function (FVC) **eFigure 7.** Caterpillar Plot for Quality of Life Outcomes **eFigure 8.** Caterpillar Plot of Physical Health Outcomes **eFigure 9.** Caterpillar Plot for Mental Health Outcomes **eTable 4.** Adverse Events Results **eTable 5.** Model Fit and Model Comparison for Adverse Events **eTable 6.** Publication Bias **eFigure 10.** Autocorrelation **eFigure 11.** Trace Plot **eTable 7.** Sensitivity Analysis **eFigure 12.** Sensitivity Analysis–Autocorrelation Plot **eFigure 13.** Sensitivity Analysis–Trace Plot

Supplement 2.
Data Sharing Statement
