# Supplementary material for: Rehabilitation Interventions for Physical Capacity and Quality of Life in Adults With Post–COVID-19 Condition: A Systematic Review and Meta-Analysis
Source: JAMA Netw Open. 2023 Sep 19;6(9):e2333838. doi: 10.1001/jamanetworkopen.2023.33838 (PMC10509723; doi:10.1001/jamanetworkopen.2023.33838)
Supplement: Supplement 1. — eAppendix. Study Protocol eTable 1. Research Strategy eMethods. Models eTable 2. Excluded Studies–Reasons for Exclusion eTable 3. Tidier eFigure 1. Caterpillar Plot for Exercise Capacity eFigure 2. Caterpillar Plot for Lower Limb Muscle Function eFigure 3. Caterpillar Plot for Dyspnea eFigure 4. Sensitivity Analysis for Dyspnea (Allocation Concealment) eFigure 5. Caterpillar Plot for Respiratory Function (FEV1) eFigure 6. Caterpillar Plot for Respiratory Function (FVC) eFigure 7. Caterpillar Plot for Quality of Life Outcomes eFigure 8. Caterpillar Plot of Physical Health Outcomes eFigure 9. Caterpillar Plot for Mental Health Outcomes eTable 4. Adverse Events Results eTable 5. Model Fit and Model Comparison for Adverse Events eTable 6. Publication Bias eFigure 10. Autocorrelation eFigure 11. Trace Plot eTable 7. Sensitivity Analysis eFigure 12. Sensitivity Analysis–Autocorrelation Plot eFigure 13. Sensitivity Analysis–Trace Plot [file jamanetwopen-e2333838-s001.pdf]

Supplemental Online Content

Pouliopoulou DV, Macdermid JC, Saunders E, et al. Rehabilitation interventions for physical capacity and quality of life in adults with post–COVID-19 condition: a systematic review and meta-analysis. *JAMA Netw Open*. 2023; 6(9):e2333838
doi:10.1001/jamanetworkopen.2023.33838

eAppendix. Study Protocol

eTable 1. Research Strategy

eMethods. Models

eTable 2. Excluded Studies–Reasons for Exclusion

eTable 3. Tidier

eFigure 1. Caterpillar Plot for Exercise Capacity

eFigure 2. Caterpillar Plot for Lower Limb Muscle Function

eFigure 3. Caterpillar Plot for Dyspnea

eFigure 4. Sensitivity Analysis for Dyspnea (Allocation Concealment)

eFigure 5. Caterpillar Plot for Respiratory Function (FEV1)

eFigure 6. Caterpillar Plot for Respiratory Function (FVC)

eFigure 7. Caterpillar Plot for Quality of Life Outcomes

eFigure 8. Caterpillar Plot of Physical Health Outcomes

eFigure 9. Caterpillar Plot for Mental Health Outcomes

eTable 4. Adverse Events Results

eTable 5. Model Fit and Model Comparison for Adverse Events

eTable 6. Publication Bias

eFigure 10. Autocorrelation

eFigure 11. Trace Plot

eTable 7. Sensitivity Analysis

eFigure 12. Sensitivity Analysis–Autocorrelation Plot

eFigure 13. Sensitivity Analysis–Trace Plot

This supplemental material has been provided by the authors to give readers additional information about their work.

eAppendix. Study Protocol

PROSPERO
International prospective register of systematic reviews

UNIVERSITY of York
Centre for Reviews and Dissemination

Systematic review

A list of fields that can be edited in an update can be found here

1. * Review title.

Give the title of the review in English

Efficacy and safety of rehabilitation interventions in patients with post-COVID syndrome: a systematic review with meta-analysis

2. Original language title.

For reviews in languages other than English, give the title in the original language. This will be displayed with the English language title.

3. * Anticipated or actual start date.

Give the date the systematic review started or is expected to start.

29/11/2022

4. * Anticipated completion date.

Give the date by which the review is expected to be completed.

28/01/2023

5. * Stage of review at time of this submission.

This field uses answers to initial screening questions. It cannot be edited until after registration.

Tick the boxes to show which review tasks have been started and which have been completed.

Update this field each time any amendments are made to a published record.

The review has not yet started: Yes

© 2023 Pouliopoulou DV et al. *JAMA Network Open.*

National Institute for Health Research

eAppendix. Study Protocol

PROSPERO
International prospective register of systematic reviews

University of York
Centre for Reviews and Dissemination

Systematic review

A list of fields that can be edited in an update can be found here

1. * Review title.

Give the title of the review in English

Efficacy and safety of rehabilitation interventions in patients with post-COVID syndrome: a systematic review with meta-analysis

2. Original language title.

For reviews in languages other than English, give the title in the original language. This will be displayed with the English language title.

3. * Anticipated or actual start date.

Give the date the systematic review started or is expected to start.

29/11/2022

4. * Anticipated completion date.

Give the date by which the review is expected to be completed.

28/01/2023

5. * Stage of review at time of this submission.

This field uses answers to initial screening questions. It cannot be edited until after registration.

Tick the boxes to show which review tasks have been started and which have been completed.

Update this field each time any amendments are made to a published record.

The review has not yet started: Yes

© 2023 Pouliopoulou DV et al. *JAMA Network Open.*

**Review stage**	**Started**	**Completed**
Preliminary searches	No	No
Piloting of the study selection process	No	No
Formal screening of search results against eligibility criteria	No	No
Data extraction	No	No
Risk of bias (quality) assessment	No	No
Data analysis	No	No

Provide any other relevant information about the stage of the review here.

6. * Named contact.

The named contact is the guarantor for the accuracy of the information in the register record. This may be any member of the review team.

Pavlos Bobos

Email salutation (e.g. "Dr Smith" or "Joanne") for correspondence:

Dr Bobos

7. * Named contact email.

Give the electronic email address of the named contact.

pbobos@uwo.ca

8. Named contact address

Give the full institutional/organisational postal address for the named contact.

1151 Richmond Street

9. Named contact phone number.

Give the telephone number for the named contact, including international dialling code.

+1-519-870-5145

10. * Organisational affiliation of the review.

Full title of the organisational affiliations for this review and website address if available. This field may be

© 2023 Pouliopoulou DV et al. *JAMA Network Open.*

Review stage	Started	Completed
Preliminary searches	No	No
Piloting of the study selection process	No	No
Formal screening of search results against eligibility criteria	No	No
Data extraction	No	No
Risk of bias (quality) assessment	No	No
Data analysis	No	No

Provide any other relevant information about the stage of the review here.

6. * Named contact.

The named contact is the guarantor for the accuracy of the information in the register record. This may be any member of the review team.

Pavlos Bobos

Email salutation (e.g. "Dr Smith" or "Joanne") for correspondence:

Dr Bobos

7. * Named contact email.

Give the electronic email address of the named contact.

pbobos@uwo.ca

8. Named contact address

Give the full institutional/organisational postal address for the named contact.

1151 Richmond Street

9. Named contact phone number.

Give the telephone number for the named contact, including international dialling code.

+1-519-870-5145

10. * Organisational affiliation of the review.

Full title of the organisational affiliations for this review and website address if available. This field may be

© 2023 Pouliopoulou DV et al. *JAMA Network Open.*

completed as 'None' if the review is not affiliated to any organisation.

Western University

Organisation web address:

11. * Review team members and their organisational affiliations.

Give the personal details and the organisational affiliations of each member of the review team. Affiliation refers to groups or organisations to which review team members belong. **NOTE: email and country now MUST be entered for each person, unless you are amending a published record.**

Ms Dimitra Vasiliki Pouliopoulou. Western University
Ms Emily Saunders. Western University
Dr Joy MacDermid. Western University
Dr Erin Miller. Western University
Dr Sue Peters. Western University
Dr Trevor Birmingham. Western University
Dr Alison Rushton. Western University
Dr Pavlos Bobos. Western University

12. * Funding sources/sponsors.

Details of the individuals, organizations, groups, companies or other legal entities who have funded or sponsored the review.

No funding was received to conduct this review

Grant number(s)

State the funder, grant or award number and the date of award

13. * Conflicts of interest.

List actual or perceived conflicts of interest (financial or academic).

None

14. Collaborators.

Give the name and affiliation of any individuals or organisations who are working on the review but who are not listed as review team members. **NOTE: email and country must be completed for each person, unless you are amending a published record.**

15. * Review question.

State the review question(s) clearly and precisely. It may be appropriate to break very broad questions down into a series of related more specific questions. Questions may be framed or refined using PI(E)COS or similar where relevant.

Are rehabilitation interventions effective at improving aerobic and respiratory function and quality of life, in

© 2023 Pouliopoulou DV et al. *JAMA Network Open.*

patients diagnosed with long-covid?

16. * Searches.

State the sources that will be searched (e.g. Medline). Give the search dates, and any restrictions (e.g. language or publication date). Do NOT enter the full search strategy (it may be provided as a link or attachment below.)

A systematic electronic search of the literature will be performed from January 2020 until December 2022, in PubMed, Scopus, CINAHL, and Clinical Trials Registry with no date restrictions. The following keywords will be used to identify potentially relevant studies: "covid-19", "long-covid", "post-covid", “sequelae”, "exercise", "exercise therapy", "rehabilitation", "physical activity", "physical therapy", "randomized controlled trials", "RCT". In addition, we will conduct a manual search of the reference lists of the included studies to identify any potential studies missed in the electronic search.

17. URL to search strategy.

Upload a file with your search strategy, or an example of a search strategy for a specific database, (including the keywords) in pdf or word format. In doing so you are consenting to the file being made publicly accessible. Or provide a URL or link to the strategy. Do NOT provide links to your search **results**.

Alternatively, upload your search strategy to CRD in pdf format. Please note that by doing so you are consenting to the file being made publicly accessible.

Do not make this file publicly available until the review is complete

18. * Condition or domain being studied.

Give a short description of the disease, condition or healthcare domain being studied in your systematic review.

The main condition being studied are patients suffering from Long covid syndrome as defined by The World Health Organization (WHO). The WHO defines Long COVID or post COVID syndrome as a case definition of “symptoms occurring three or more months after acute SARS-CoV-2 infection that last for at least two months and is not explainable by an alternative condition”.

19. * Participants/population.

Specify the participants or populations being studied in the review. The preferred format includes details of both inclusion and exclusion criteria.

We will include randomized trials that include patients with Long COVID syndrome as defined by The World Health Organization (WHO). Studies that include interventions during acute care will be excluded.

20. * Intervention(s), exposure(s).

Give full and clear descriptions or definitions of the interventions or the exposures to be reviewed. The preferred format includes details of both inclusion and exclusion criteria.

We will include studies that used aerobic, strength, conditioning and respiratory exercise interventions and treatment modalities. Studies that use a medication-only treatment will be excluded.

21. * Comparator(s)/control.

Where relevant, give details of the alternatives against which the intervention/exposure will be compared (e.g. another intervention or a non-exposed control group). The preferred format includes details of both inclusion and exclusion criteria.

The interventions in the control groups can be either usual/standard care or waitlist/control.

22. * Types of study to be included.

Give details of the study designs (e.g. RCT) that are eligible for inclusion in the review. The preferred format includes both inclusion and exclusion criteria. If there are no restrictions on the types of study, this should be stated.

We will only include randomised control trials. Studies that are self-controlled and do not include a control group will be excluded. Non-randomised, quasi-experimental and feasibility trials will be excluded. We will also exclude observational studies, case series, case reports and pilot studies.

23. Context.

Give summary details of the setting or other relevant characteristics, which help define the inclusion or exclusion criteria.

24. * Main outcome(s).

Give the pre-specified main (most important) outcomes of the review, including details of how the outcome is defined and measured and when these measurement are made, if these are part of the review inclusion criteria.

The main outcome will be performance-based aerobic function, measured with the 6-minute walking test. If similar measurements are used instead, such as a 3min-walking test or a 2-min walking test, we will extract the outcome measures and pool them using Standardised Mean Difference. The timepoint of the primary outcome will be directly post-intervention.

Measures of effect

Please specify the effect measure(s) for you main outcome(s) e.g. relative risks, odds ratios, risk difference, and/or 'number needed to treat.

The effect measures for the main outsome will be the standardised mean differences (SMD) of change scores with 95% credible intervals. We will use OpenBUGS and STATA (Stata Statistical Software: Release 16, StataCorp LLC) to perform the meta-analysis.

25. * Additional outcome(s).

© 2023 Pouliopoulou DV et al. *JAMA Network Open.*

List the pre-specified additional outcomes of the review, with a similar level of detail to that required for main outcomes. Where there are no additional outcomes please state 'None' or 'Not applicable' as appropriate to the review

We will include long term performance-based aerobic function measured by the 6-minute walking test in our secondary outcomes. We will also include performance-based aerobic function measured by the 30-sec sit and stand test in both short and long-term follow-ups in our secondary outcomes. Other secondary outcomes will be patient-reported function, performance-based and patient reported respiratory function, Global Health Status and patient reported quality of life both in short and in long term follow-ups. If more than one effect measures are presented in a study for the same outcome, we will extract the one reported as the primary outcome in the study. We will also monitor adverse events and serious adverse events related to the interventions across all studies. Adverse events and non-adverse events unrelated to the interventions will not be included.

Measures of effect

Please specify the effect measure(s) for you additional outcome(s) e.g. relative risks, odds ratios, risk difference, and/or 'number needed to treat.

The effect measures for performance-based and patient-reported aerobic function, respiratory function, Global Health Status and quality of life (continuous outcomes) will be the standardised mean differences (SMD) of change scores with 95% credible intervals. We will use OpenBUGS and STATA (Stata Statistical Software: Release 16, StataCorp LLC) to perform a meta-analysis using the standardised mean difference with 95% credible intervals. The effect measured for the adverse events (dichotomous outcome) will be the risk ratios with 95% credible intervals (CrIs).

26. * Data extraction (selection and coding).

Describe how studies will be selected for inclusion. State what data will be extracted or obtained. State how this will be done and recorded.

The selection of individual studies will involve two independent reviewers who will perform a systematic electronic search of the databases. Two reviewers will identify potentially relevant articles, remove duplicates, and then screen titles and abstracts. The full text of any study marked include or uncertain will be obtained and the eligibility criteria will be applied. Two independent researchers will extract the data from the eligible included studies. Data extraction will include study, author, year, sample characteristics, intervention, comparison groups, aerobic function, respiratory function, Global Health Status, quality of life, and adverse or serious adverse events.

27. * Risk of bias (quality) assessment.

State which characteristics of the studies will be assessed and/or any formal risk of bias/quality assessment tools that will be used.

© 2023 Pouliopoulou DV et al. *JAMA Network Open.*

Two independent authors will assess the included RCTs for risk of bias (ROB). The ROB assessment will be performed using the Cochrane Risk of Bias tool. The ROB tool is based on 7 items, random sequence generation, allocation concealment, blinding of participants and personnel, blinding of outcome assessment, incomplete outcome data, selective reporting, and other bias. The adequacy of each of the seven ROB domains will be rated as “low,” “unclear,” or “high” risk. We will summarize the assessment of ROB per study as Low ROB (if low ROB was judged for all the seven domains); as Unclear ROB (if unclear ROB was judged for one or more of the seven domains); and as High ROB (if high ROB was judged for one or more of the seven domains). The GRADE guidelines will be used to evaluate the certainty of evidence. The GRADE approach includes assessing ROB for study limitations, inconsistency, publication bias, imprecision, and indirectness. The rating of the quality of outcome across trials will be carried out to summarize the extent of our confidence (high, moderate, low, or very low).

28. * Strategy for data synthesis.

Describe the methods you plan to use to synthesise data. This **must not be generic text** but should be **specific to your review** and describe how the proposed approach will be applied to your data. If meta-analysis is planned, describe the models to be used, methods to explore statistical heterogeneity, and software package to be used.

For data synthesis we will use OpenBUGS (version 3.2.3, Cambridge, UK) and STATA (Stata Statistical Software: Release 16, StataCorp LLC). We will perform a pair-wise Bayesian random-effects meta-analyses using the normal likelihood to compare rehab interventions versus usual care, waitlist or no intervention using standardized mean difference with 95% credible intervals for continuous outcomes and risk ratio with 95% credible Intervals for binary ones. If meta-analysis is not possible, we will present the results in a narrative format. We will provide descriptive statistics of the patient groups, interventions, outcomes, and adverse effects of treatments. We will report all results using the intention-to-treat principle (the sample randomized in the study). If trials report standard error, we will transform it into standard deviation. For missing values such as standard deviation, we will use the following methods: 1. We convert 95% Confidence Intervals or Standard Error to Standard Deviation; 2. Digitizer software — Digitize a scanned graph or chart into (x, y)-data, will be used when data was presented in graphic form (http://www.digitizeit.de/). To facilitate analysis, data imputation rules will be used when necessary. We will assess small study effects with Harbord test and we will investigate the presence of publication bias with funnel plots. In the presence of publication bias we will impute the missing studies to account for publication bias in the meta-analysis. We will compare the observed and the imputed studies by using the non-parametric “trim and fill” method. We will use contour-enhanced funnel plot to assess if imputed studies fall in the region of statistical significance.

29. * Analysis of subgroups or subsets.

© 2023 Pouliopoulou DV et al. *JAMA Network Open.*

State any planned investigation of ‘subgroups’. Be clear and specific about which type of study or participant will be included in each group or covariate investigated. State the planned analytic approach.

Heterogeneity will be assessed with tau squared statistic (?2). In the presence of substantial heterogeneity in the meta-analysis, we planned to perform separate univariate meta-regressions to quantify factors that may contribute to substantial heterogeneity. We prespecified the following factors: allocation concealment, time of follow-up and length of inpatient stay.

30. * Type and method of review.

Select the type of review, review method and health area from the lists below.

Type of review

Cost effectiveness

No

Diagnostic

No

Epidemiologic

No

Individual patient data (IPD) meta-analysis

No

Intervention

No

Living systematic review

No

Meta-analysis

Yes

Methodology

No

Narrative synthesis

No

Network meta-analysis

No

Pre-clinical

No

Prevention

No

Prognostic

No

Prospective meta-analysis (PMA)

© 2023 Pouliopoulou DV et al. *JAMA Network Open.*

No

Review of reviews
No

Service delivery
No

Synthesis of qualitative studies
No

Systematic review
Yes

Other
No

Health area of the review

Alcohol/substance misuse/abuse
No

Blood and immune system
No

Cancer
No

Cardiovascular
Yes

Care of the elderly
No

Child health
No

Complementary therapies
No

COVID-19
Yes

For COVID-19 registrations please tick all categories that apply. Doing so will enable your record to appear in area-specific searches

Chinese medicine
Diagnosis
Epidemiological
Genetics
Health impacts
Immunity

© 2023 Pouliopoulou DV et al. *JAMA Network Open.*

Long COVID
Mental health
PPE
Prognosis
Public health intervention
Rehabilitation
Service delivery
Transmission
Treatments
Vaccines
Other

Crime and justice
No

Dental
No

Digestive system
No

Ear, nose and throat
No

Education
No

Endocrine and metabolic disorders
No

Eye disorders
No

General interest
No

Genetics
No

Health inequalities/health equity
No

Infections and infestations
Yes

International development
No

Mental health and behavioural conditions
No

Musculoskeletal
No

© 2023 Pouliopoulou DV et al. *JAMA Network Open.*

Neurological

No

Nursing

No

Obstetrics and gynaecology

No

Oral health

No

Palliative care

No

Perioperative care

No

Physiotherapy

Yes

Pregnancy and childbirth

No

Public health (including social determinants of health)

No

Rehabilitation

Yes

Respiratory disorders

Yes

Service delivery

No

Skin disorders

No

Social care

No

Surgery

No

Tropical Medicine

No

Urological

No

Wounds, injuries and accidents

No

Violence and abuse

© 2023 Pouliopoulou DV et al. *JAMA Network Open.*

No

31. Language.

Select each language individually to add it to the list below, use the bin icon to remove any added in error.
English

There is not an English language summary

32. * Country.

Select the country in which the review is being carried out. For multi-national collaborations select all the countries involved.

Canada

33. Other registration details.

Name any other organisation where the systematic review title or protocol is registered (e.g. Campbell, or The Joanna Briggs Institute) together with any unique identification number assigned by them. If extracted data will be stored and made available through a repository such as the Systematic Review Data Repository (SRDR), details and a link should be included here. If none, leave blank.

34. Reference and/or URL for published protocol.

If the protocol for this review is published provide details (authors, title and journal details, preferably in Vancouver format)

Add web link to the published protocol.

Or, upload your published protocol here in pdf format. Note that the upload will be publicly accessible.

No I do not make this file publicly available until the review is complete

Please note that the information required in the PROSPERO registration form must be completed in full even if access to a protocol is given.

35. Dissemination plans.

Do you intend to publish the review on completion?

Yes

Give brief details of plans for communicating review findings.?

© 2023 Pouliopoulou DV et al. *JAMA Network Open.*

36. Keywords.

Give words or phrases that best describe the review. Separate keywords with a semicolon or new line. Keywords help PROSPERO users find your review (keywords do not appear in the public record but are included in searches). Be as specific and precise as possible. Avoid acronyms and abbreviations unless these are in wide use.

37. Details of any existing review of the same topic by the same authors.

If you are registering an update of an existing review give details of the earlier versions and include a full bibliographic reference, if available.

38. * Current review status.

Update review status when the review is completed and when it is published.New registrations must be ongoing so this field is not editable for initial submission.

Please provide anticipated publication date

Review_Ongoing

39. Any additional information.

Provide any other information relevant to the registration of this review.

40. Details of final report/publication(s) or preprints if available.

Leave empty until publication details are available OR you have a link to a preprint (NOTE: this field is not editable for initial submission). List authors, title and journal details preferably in Vancouver format.

Give the link to the published review or preprint.

© 2023 Pouliopoulou DV et al. *JAMA Network Open*.

References

1. Abodonya AM, Abdelbasset WK, Awad EA, Elalfy IE, Salem HA, Elsayed SH. Inspiratory muscle training for recovered COVID-19 patients after weaning from mechanical ventilation: A pilot control clinical study. Medicine 2021;100(13).

2. Achabaeva AB, Kaisinova AS, Gusov RM, Taimazova ZA, Efimenko NV, Chalaya EN. [Natural therapeutic factors in medical rehabilitation of patients with post-COVID syndrome]. Voprosy kurortologii, fizioterapii, i lechebnoi fizicheskoi kultury 2022;99(6. Vyp. 2):5–11.

3. Ahmed I, Inam AB, Belli S, Ahmad J, Khalil W, Jafar MM. Effectiveness of aerobic exercise training program on cardio-respiratory fitness and quality of life in patients recovered from COVID-19. European Journal of Physiotherapy 2021;1–6.

4. Al-Aly Z, Xie Y, Bowe B. High-dimensional characterization of post-acute sequelae of COVID-19. Nature 2021;594(7862):259–64.

5. Al Chikhanie Y, Veale D, Schoeffler M, Pépin JL, Verges S, Hérengt F. Effectiveness of pulmonary rehabilitation in COVID-19 respiratory failure patients post-ICU. Respiratory physiology & neurobiology 2021;287:103639.

6. Besnier F, Bérubé B, Malo J, et al. Cardiopulmonary Rehabilitation in Long-COVID-19 Patients with Persistent Breathlessness and Fatigue: The COVID-Rehab Study. International Journal of Environmental Research and Public Health 2022;19(7):4133.

7. Betschart M, Rezek S, Unger I, et al. Feasibility of an outpatient training program after COVID-19. International journal of environmental research and public health 2021;18(8):3978.

8. Bodrova RA, Kuchumova TV, Zakamyrdina AD, Yunusova ER, Fadeev GY. [Efficacy of Low-Frequency Magnetic Therapy in Patients with COVID-19 Pneumonia]. Vopr Kurortol Fizioter Lech Fiz Kult 2020;97(6):11–6.

9. Bragin M, Goloborodko E, Erofeev G, et al. The use of bioacoustic stimulation of the respiratory system in complex medical rehabilitation at a health resort facility for patients who have had covid-associated pneumonia. Open Access Macedonian Journal of Medical Sciences 2021;9:1607–14.

10. Brame J, Centner C, Berg N, Bartlam M, Gollhofer A, König D. Effects of a 12-Week Web-Based Weight Loss Program for Adults With Overweight and Obesity on COVIDAge and Lifestyle-Related Cardiometabolic Risk Factors: A Randomized Controlled Trial. Front Public Health 2022;10:868255.

11. Cahalan RM, Meade C, Mockler S. SingStrong--A singing and breathing retraining intervention for respiratory and other common symptoms of long COVID: A pilot study. Canadian Journal of Respiratory Therapy 2022;58:20–7.

12. Calabrese M, Garofano M, Palumbo R, et al. Exercise training and cardiac rehabilitation in COVID-19 patients with cardiovascular complications: state of art. Life 2021;11(3):259.

© 2023 Pouliopoulou DV et al. *JAMA Network Open*.

13. Calvo-Paniagua J, Díaz-Arribas MJ, Valera-Calero JA, et al. A tele-health primary care rehabilitation program improves self-perceived exertion in COVID-19 survivors experiencing Post-COVID fatigue and dyspnea: A quasi-experimental study. PloS one 2022;17(8):e0271802.

14. Corna S, Giardini M, Godi M, Bellotti L, Arcolin I. Effects of Aerobic Training in Patients with Subacute COVID-19: A Randomized Controlled Feasibility Trial. International journal of environmental research and public health 2022;19(24).

15. Dalbosco-Salas M, Torres-Castro R, Rojas Leyton A, et al. Effectiveness of a primary care telerehabilitation program for post-COVID-19 patients: A feasibility study. Journal of clinical medicine 2021;10(19):4428.

16. Eross B, Molnár Z, Szakács Z, et al. Personalised health education against health damage of COVID-19 epidemic in the elderly Hungarian population (PROACTIVE-19): Protocol of an adaptive randomised controlled clinical trial. Trials [Internet] 2020;21(1). Available from: https://www.scopus.com/inward/record.uri?eid=2-s2.0-85092297411&doi=10.1186%2fs13063-020-04733-0&partnerID=40&md5=75078a2764a4a96806142c7ff1f16126

17. Gloeckl R, Leitl D, Jarosch I, et al. Pulmonary rehabilitation in long COVID: more than just natural recovery!? ERJ Open Research 2021;7(3).

18. Hawkins J, Hires C, Keenan L, Dunne E. Aromatherapy blend of thyme, orange, clove bud, and frankincense boosts energy levels in post-COVID-19 female patients: A randomized, double-blinded, placebo controlled clinical trial. Complementary Therapies in Medicine 2022;67:102823.

19. Hermann M, Pekacka-Egli A-M, Witassek F, Baumgaertner R, Schoendorf S, Spielmanns M. Feasibility and efficacy of cardiopulmonary rehabilitation following COVID-19. American journal of physical medicine & rehabilitation 2020;

20. Kaisinova A, Achabaeva A, Starokozhko L, Gaydamaka I, Kaisinova E, Kazakov V. [NATURAL THERAPEUTIC FACTORS IN MEDICAL REHABILITATION OF PATIENTS WITH POST-COVID-19 AT OUTPATIENT TREATMENT STAGE]. Georgian Med News 2021;(318):110–4.

21. Kim K-H, Kim D-H. Effects of Maitland Thoracic Joint Mobilization and Lumbar Stabilization Exercise on Diaphragm Thickness and Respiratory Function in Patients with a History of COVID-19. International journal of environmental research and public health 2022;19(24).

22. Kortianou EA, Tsimouris D, Mavronasou A, et al. Application of a home-based exercise program combined with tele-rehabilitation in previously hospitalized patients with COVID-19: A feasibility, single-cohort interventional study. Pneumon 2022;35(2):12.

23. Kuut T, Müller F, Aldenkamp A, et al. A randomised controlled trial testing the efficacy of Fit after COVID, a cognitive behavioural therapy targeting severe post-infectious fatigue following COVID-19 (ReCOVer): study protocol. Trials 2021;22(1):1–15.

24. Li J, Li X, Jiang J, et al. The effect of cognitive behavioral therapy on depression, anxiety, and stress in patients with COVID-19: a randomized controlled trial. Frontiers in psychiatry 2020;11:580827.

© 2023 Pouliopoulou DV et al. *JAMA Network Open*.

25. Liu Z, Qiao D, Xu Y, et al. The efficacy of computerized cognitive behavioral therapy for depressive and anxiety symptoms in patients with COVID-19: randomized controlled trial. Journal of medical Internet research 2021;23(5):e26883.

26. Liu S, Zhan C, Ma Y, et al. Effect of qigong exercise and acupressure rehabilitation program on pulmonary function and respiratory symptoms in patients hospitalized with severe COVID-19: a randomized controlled trial. Integrative Medicine Research 2021;10:100796.

27. Llurda-Almuzara L, Rodriguez-Sanz J, Lopez-de-Celis C, et al. Effects of Adding an Online Exercise Program on Physical Function in Individuals Hospitalized by COVID-19: A Randomized Controlled Trial. International journal of environmental research and public health 2022;19(24).

28. Lu ZH, Yang CL, Yang GG, et al. Efficacy of the combination of modern medicine and traditional Chinese medicine in pulmonary fibrosis arising as a sequelae in convalescent COVID-19 patients: a randomized multicenter trial. Infect Dis Poverty 2021;10(1):31.

29. Luo ZH, Wang KX, Zhang YL, et al. [Thumb-tack needles based on “Biaoben acupoint compatibility” for sequela of COVID-19 during recovery period]. Zhongguo Zhen Jiu 2022;42(3):281–6.

30. Martin I, Braem F, Baudet L, et al. Follow-up of functional exercise capacity in patients with COVID-19: It is improved by telerehabilitation. Respiratory medicine 2021;183(8908438, rme):106438.

31. Mayer KP, Parry SM, Kalema AG, et al. Safety and feasibility of an interdisciplinary treatment approach to optimize recovery from critical coronavirus disease 2019. Critical care explorations 2021;3(8).

32. Michler E, Dolzhenko Y, Altmann C. [Late neurological consequences of a SARS-CoV-2 infection]. Dtsch Med Wochenschr 2022;147(4):173–7.

33. Mukand JA. RIMJ Special Section: The Evaluation & Rehabilitation of Long COVID. R I Med J (2013) 2022;105(7):7–10.

34. NAGY EN, ELIMY DA, ALI AY, EZZELREGAL HG, ELSAYED MM. INFLUENCE OF MANUAL DIAPHRAGM RELEASE TECHNIQUE COMBINED WITH INSPIRATORY MUSCLE TRAINING ON SELECTED PERSISTENT SYMPTOMS IN MEN WITH POST-COVID-19 SYNDROME: A RANDOMIZED CONTROLLED TRIAL. Journal of Rehabilitation Medicine (Stiftelsen Rehabiliteringsinformation) 2022;54:1–10.

35. Nambi G, Abdelbasset WK, Alrawaili SM, et al. Comparative effectiveness study of low versus high-intensity aerobic training with resistance training in community-dwelling older men with post-COVID 19 sarcopenia: A randomized controlled trial. Clinical rehabilitation 2022;36(1):59–68.

36. Orlova EV, Lyamina NP, Skorobogatyth NV, Pogonchenkova IV. Clinical Efficacy of Individually Dosed Intermittent Hypoxia-Hyperoxic Therapy in Osteoarthritis Patients with Post-Covid Syndrome. Vestnik Vosstanovitel’noj Mediciny 2022;21(2):6–16.

37. Palau P, Dominguez E, Gonzalez C, et al. Effect of a home-based inspiratory muscle training programme on functional capacity in postdischarged patients with long COVID: the InsCOVID trial. BMJ open respiratory research 2022;9(1).

© 2023 Pouliopoulou DV et al. *JAMA Network Open*.

38. Pehlivan E, Palalı İ, Atan SG, Turan D, Çınarka H, Çetinkaya E. The effectiveness of POST-DISCHARGE telerehabilitation practices in COVID-19 patients: Tele-COVID study-randomized controlled trial. Annals of Thoracic Medicine 2022;17(2):110.

39. Piquet V, Luczak C, Seiler F, et al. Do patients with COVID-19 benefit from rehabilitation? Functional outcomes of the first 100 patients in a COVID-19 rehabilitation unit. Archives of Physical Medicine and Rehabilitation 2021;102(6):1067–74.

40. Plaza M de la, Morales MB, Sevilla GGP de, Flor ÁG de la, Morales CR, Rubio M. Telematics program of breathing exercises and mindfulness for post-coronavirus disease 2019 patients. Revista da Associação Médica Brasileira 2022;68:632–5.

41. Sharma P, Goswami SK. Pulmonary Tele-Rehabilitation in Patients (Post Covid-19) With Respiratory Complications: A Randomized Controlled Trial. Editorial Advisory Board 2022;16(2):182.

42. Shogenova LV, Tuet TT, Kryukova NO, et al. KalHydrogen inhalation in rehabilitation program of the medical staff recovered from COVID-19. Cardiovascular Therapy and Prevention (Russian Federation) 2021;20(6):24–32.

43. Spruit MA, Holland AE, Singh SJ, Tonia T, Wilson KC, Troosters T. COVID-19: interim guidance on rehabilitation in the hospital and post-hospital phase from a European Respiratory Society-and American Thoracic Society-coordinated international task force. European respiratory journal 2020;56(6).

44. Srinivasan V, Kandakurti P, Suganthirababu P, Jebasingh K. EFFICACY OF PURSED LIP BREATHING WITH BHASTRIKA PRANAYAMA VS INCENTIVE SPIROMETRY IN REHABILITATING POST COVID 19 FOLLOW UP-A RANDOMIZED CONTROL STUDY. Türk Fizyoterapi ve Rehabilitasyon Dergisi/Turkish Journal of Physiotherapy and Rehabilitation 2021;32:402.

45. Szczegielniak J, Bogacz K, Majorczyk E, Szczegielniak A, Luniewski J. Post-COVID-19 rehabilitation - a Polish pilot program. Medycyna pracy 2021;72(5):611–6.

46. Tang Y, Jiang J, Shen P, et al. Liuzijue is a promising exercise option for rehabilitating discharged COVID-19 patients. Medicine 2021;100(6).

47. Toulgui E, Benzarti W, Rahmani C, et al. Impact of cardiorespiratory rehabilitation program on submaximal exercise capacity of Tunisian male patients with post-COVID19: A pilot study. Frontiers in physiology 2022;13(101549006):1029766.

48. Tsutsui M, Gerayeli F, Sin DD. Pulmonary rehabilitation in a post-COVID-19 world: telerehabilitation as a new standard in patients with COPD. International journal of chronic obstructive pulmonary disease 2021;16:379.

49. Udina C, Ars J, Morandi A, Vilaró J, Cáceres C, Inzitari M. Rehabilitation in adult post-COVID-19 patients in post-acute care with therapeutic exercise. The Journal of frailty & aging 2021;10(3):297–300.

© 2023 Pouliopoulou DV et al. *JAMA Network Open*.

50. Vallier J-M, Simon C, Bronstein A, et al. Randomized controlled trial of home-based vs. hospital-based pulmonary rehabilitation in post COVID-19 patients. European journal of physical and rehabilitation medicine 2023;(101465662).

51. Vink M, Vink-Niese A. Could cognitive behavioural therapy be an effective treatment for long COVID and post COVID-19 fatigue syndrome? lessons from the Qure study for Q-fever fatigue syndrome. MDPI; 2020. p. 552.

52. Wang F, Kream RM, Stefano GB. Long-term respiratory and neurological sequelae of COVID-19. Medical science monitor: international medical journal of experimental and clinical research 2020;26:e928996-1.

53. Xiong W, Kwan P, Zhou D, Del Felice A, Duncan JS, Sander JW. Acute and late neurological complications of COVID19: the quest for evidence. Brain 2020;143(12):e99.

54. Zilberman-Itskovich S, Catalogna M, Sasson E, et al. Hyperbaric oxygen therapy improves neurocognitive functions and symptoms of post-COVID condition: randomized controlled trial. Sci Rep 2022;12(1):11252.

55. do Amaral VT, Viana AA, Heubel AD, et al. Cardiovascular, Respiratory, and Functional Effects of Home-based Exercise Training after COVID-19 Hospitalization. Med Sci Sports Exerc 2022;

56. Capin JJ, Jolley SE, Morrow M, et al. Safety, feasibility and initial efficacy of an app-facilitated telerehabilitation (AFTER) programme for COVID-19 survivors: a pilot randomised study. BMJ open 2022;12(7):e061285.

57. Del Corral T, Fabero-Garrido R, Plaza-Manzano G, Fernandez-de-Las-Penas C, Navarro-Santana M, Lopez-de-Uralde-Villanueva I. Home-based respiratory muscle training on quality of life and exercise tolerance in long-term post-COVID-19: Randomized controlled trial. Annals of physical and rehabilitation medicine 2022;66(1):101709.

58. De Souza Y, Macedo J, Nascimento R, et al. Low-Intensity Pulmonary Rehabilitation Through Videoconference for Post-Acute COVID-19 Patients. In: TP101. TP101 REHABILITATION IN THE TIME OF COVID-19. American Thoracic Society; 2021. p. A4124–A4124.

59. Jimeno-Almazan A, Buendia-Romero A, Martinez-Cava A, et al. Effects of a concurrent training, respiratory muscle exercise, and self-management recommendations on recovery from post-COVID-19 conditions: the RECOVE trial. Journal of applied physiology (Bethesda, Md : 1985) 2023;134(1):95–104.

60. Jimeno-Almazan A, Franco-Lopez F, Buendia-Romero A, et al. Rehabilitation for post-COVID-19 condition through a supervised exercise intervention: A randomized controlled trial. Scandinavian journal of medicine & science in sports 2022;32(12):1791–801.

61. Li J, Xia W, Zhan C, et al. Effectiveness of a telerehabilitation program for COVID-19 survivors (TERECO) on exercise capacity, pulmonary function, lower limb muscle strength, and quality of life: a randomized controlled trial. medRxiv 2021;

© 2023 Pouliopoulou DV et al. *JAMA Network Open*.

62. Liu K, Zhang W, Yang Y, Zhang J, Li Y, Chen Y. Respiratory rehabilitation in elderly patients with COVID-19: A randomized controlled study. Complementary therapies in clinical practice 2020;39:101166.

63. McNarry MA, Berg RM, Shelley J, et al. Inspiratory Muscle Training Enhances Recovery Post COVID-19: A Randomised Controlled Trial. European Respiratory Journal 2022;

64. Okan F, Okan S, Duran Yücesoy F. Evaluating the Efficiency of Breathing Exercises via Telemedicine in Post-Covid-19 Patients: Randomized Controlled Study. Clinical Nursing Research 2022;10547738221097240.

65. Philip KE, Owles H, McVey S, et al. An online breathing and wellbeing programme (ENO Breathe) for people with persistent symptoms following COVID-19: a parallel-group, single-blind, randomised controlled trial. The Lancet Respiratory Medicine 2022;

66. Rodriguez-Blanco C, Bernal-Utrera C, Anarte-Lazo E, Gonzalez-Gerez JJ, Saavedra-Hernandez M. A 14-Day Therapeutic Exercise Telerehabilitation Protocol of Physiotherapy Is Effective in Non-Hospitalized Post-COVID-19 Conditions: A Randomized Controlled Trial. JCM 2023;12(3):776.

67. Romanet C, Wormser J, Fels A, et al. Effectiveness of endurance training rehabilitation after hospitalisation in intensive care for COVID-19-related acute respiratory distress syndrome on dyspnoea (RECOVER): a randomised controlled, open-label multicentre trial. medRxiv 2022;

68. SARI F, BAYRAM S, PALA GG, COMCE F, KUCUK H, OSKAY D. Effects of Inspiratory Muscle Training in Patients with post-COVID-19. Journal of Harran University Medical Faculty 2022;19(3):581–8.

69. Sahin H, Naz I, Karadeniz G, Sunecli O, Polat G, Ediboglu O. Effects of a home-based pulmonary rehabilitation program with and without telecoaching on health-related outcomes in COVID-19 survivors: a randomized controlled clinical study. Jornal brasileiro de pneumologia : publicacao oficial da Sociedade Brasileira de Pneumologia e Tisilogia 2023;49(1):e20220107.

© 2023 Pouliopoulou DV et al. *JAMA Network Open*.

eTable 1. Research strategy

MEDLINE, Feb 10th 2023

**1**	**COVID-19/ or SARS-CoV-2/**	**215156**
**2**	"Physical and Rehabilitation Medicine"/ or Rehabilitation Research/ or Rehabilitation/	21606
**3**	("long covid*" or "post covid*" or "long-covid*" or "post-covid*" or "covid* sequela*").tw.	7677
**4**	Physical Therapy Modalities/	40348
**5**	Exercise Therapy/	48171
**6**	2 or 4 or 5	105687
**7**	("physical therapy" or "physiotherapy" or "rehabilitation" or "exercise").tw.	504861
**8**	6 or 7	545923
**9**	1 or 3	218382
**10**	8 and 9	4194
**11**	Randomized Controlled Trial/ or Clinical Trial/	908100
**12**	("RCT" or "randomised control* trial" or "randomized control* trial" or "feasibility" or "pilot" or "trial").tw.	1764982
**13**	11 or 12	1764982
**14**	10 and 13	508

© 2023 Pouliopoulou DV et al. *JAMA Network Open.*

eMethods. Models

eBUGS code. Fixed and random-effects model

Fixed-effect model

```
model {
            for(i in 1:ns)
            {
            mu[i] ~ dnorm(0,.0001)
            for (k in 1:2) {
                  r[i,k] ~ dbin(p[i,k],n[i,k])
                  logit(p[i,k]) <- mu[i] + d[k]
            }
      }
d[1]<- 0
d[2] ~ dnorm(0,.0001)
or <- exp(d[2])
prob.harm <- step(d[2])
}
```

Random-effects Model

```
model {
for(i in 1:ns) {
      delta[i,1] <- 0
      mu[i] ~ dnorm(0,.0001)
      for (k in 1:2) {
                  r[i,k] ~ dbin(p[i,k],n[i,k])
                  logit(p[i,k]) <- mu[i] + delta[i,k]
      }
      delta[i,2] ~ dnorm(d[2], tau)
}
d[1]<- 0
d[2] ~ dnorm(0,.0001)
sd ~ dunif(0,2)
tau <- pow(sd,-2)
or <- exp(d[2])
prob.harm <- step(d[2])
}
```

© 2023 Pouliopoulou DV et al. *JAMA Network Open.*

eTable 2. Excluded studies – reasons for exclusion

Study	Reason for exclusion
**Abodonya 2021**[1]	Wrong study design
**Achabaeva 2022**[2]	Wrong intervention
**Ahmed 2021**[3]	Wrong study design
**Al-Aly 2021**[4]	Wrong study design
**Al Chikhanie 2021**[5]	Wrong study design
**Besnier 2022**[6]	protocol only
**Betschart 2021**[7]	Wrong study design
**Bodrova 2020**[8]	Wrong intervention
**Bragin 2021**[9]	Wrong intervention
**Brame 2022**[10]	Wrong patient population
**Cahalan 2022**[11]	Wrong study design
**Calabrese 2021**[12]	Wrong study design
**Calvo-Paniagua 2022**[13]	Wrong study design
**Corna 2022**[14]	Wrong patient population
**Dalbosco-Salas 2021**[15]	Wrong study design
**Eross 2020**[16]	Wrong study design
**Gloeckl 2021**[17]	Wrong study design
**Hawkins 2022**[18]	Wrong intervention
**Hermann 2020**[19]	Wrong study design
**Kaisinova 2021**[20]	full text not available
**Kim 2022**[21]	Wrong comparator
**Kortianou 2022**[22]	Wrong study design
**Kuut 2021**[23]	protocol only
**Li 2020**[24]	Wrong patient population
**Liu 2021**[25]	Wrong intervention
**Liu 2021**[26]	Wrong patient population
**Llurda-Almuzara 2022**[27]	Wrong patient population
**Lu 2021**[28]	Wrong intervention
**Luo 2022**[29]	Wrong intervention
**Martin 2021**[30]	Wrong study design
**Mayer 2021**[31]	Wrong study design
**Michler 2022**[32]	Wrong study design
**Mukand 2022**[33]	Wrong study design
**Nagy 2022**[34]	Wrong comparator
**Nambi 2022**[35]	Wrong comparator
**Orlova 2022**[36]	Wrong intervention
**Palau 2022**[37]	missing data
**Pehlivan 2022**[38]	missing data
**Piquet 2021**[39]	Wrong study design
**Plaza 2022**[40]	Wrong study design
**Sharma 2022**[41]	missing data
**Shogenova 2021**[42]	Wrong patient population
**Spruit 2020**[43]	Wrong study design
**Srinivasan 2021**[44]	Wrong intervention
**Szczegielniak 2021**[45]	Wrong study design
**Tang 2021**[46]	Wrong study design
**Toulgui 2022**[47]	Wrong study design
**Tsutsui 2021**[48]	Wrong study design
**Udina 2021**[49]	Wrong study design
**Vallier 2023**[50]	Wrong comparator

© 2023 Pouliopoulou DV et al. *JAMA Network Open.*

**Vink 2020[51]**	Wrong study design
**Wang 2020[52]**	Wrong study design
**Xiong 2020[53]**	Wrong study design
**Zilberman-Itskovich 2022[54]**	Wrong intervention

© 2023 Pouliopoulou DV et al. *JAMA Network Open.*

eTable 3. Tidier

	TiDiER checklist item											
**Author**	**1**	**2**	**3**	**4**	**5**	**6**	**7**	**8**	**9**	**10**	**11**	**12**
Amaral 2022[55]	✓	✓	✓	✓	✓	✓	✓	✓	✓	NA	x	x
Capin 2022[56]	✓	✓	✓	✓	✓	✓	✓	x	x	NA	✓	✓
de Corral 2022[57]	✓	✓	✓	✓	✓	✓	✓	✓	✓	NA	x	x
de Souza[58]	✓	✓	x	x	x	x	✓	x	x	NA	x	x
Jimeno-Almazan 2022[59,60]	✓	✓	x	✓	✓	✓	✓	✓	✓	NA	✓	x
Li 2022[61]	✓	✓	x	x	✓	✓	✓	x	✓	NA	x	x
Liu 2022[62]	✓	✓	✓	✓	x	✓	x	✓	✓	NA	x	x
McNarry 2022[63]	✓	✓	✓	✓	x	✓	✓	✓	✓	NA	x	✓
Okan 2022[64]	✓	✓	✓	✓	✓	✓	✓	✓	✓	NA	x	✓
Phillip 2022[65]	✓	✓	✓	✓	✓	✓	✓	✓	x	NA	x	✓
Rodriguez-Blanco 2023[66]	✓	✓	✓	✓	✓	✓	✓	✓	x	NA	x	x
Romanet 2022[67]	✓	✓	x	✓	✓	x	x	✓	x	NA	x	✓
Sari 2023[68]	✓	✓	✓	✓	✓	✓	✓	✓	✓	NA	x	✓
Sahin 2023[69]	✓	✓	✓	✓	✓	✓	✓	✓	✓	NA	✓	✓

1. Brief name
2. Why (rationale, theory, goal)
3. Materials used in intervention (physical or informational)
4. Procedures (activities, processes)
5. Intervention providers
6. Modes of delivery (e.g. face-to-face, virtual, individual or group)
7. Location of intervention
8. When and how much (number of sessions, time period, schedule and dose)
9. Details of tailoring
10. Modifications to intervention
11. Planned adherence and fidelity assessment
12. Actual adherence and fidelity

NA: not applied

© 2023 Pouliopoulou DV et al. *JAMA Network Open.*

eFigure 1. Caterpillar plot for exercise capacity

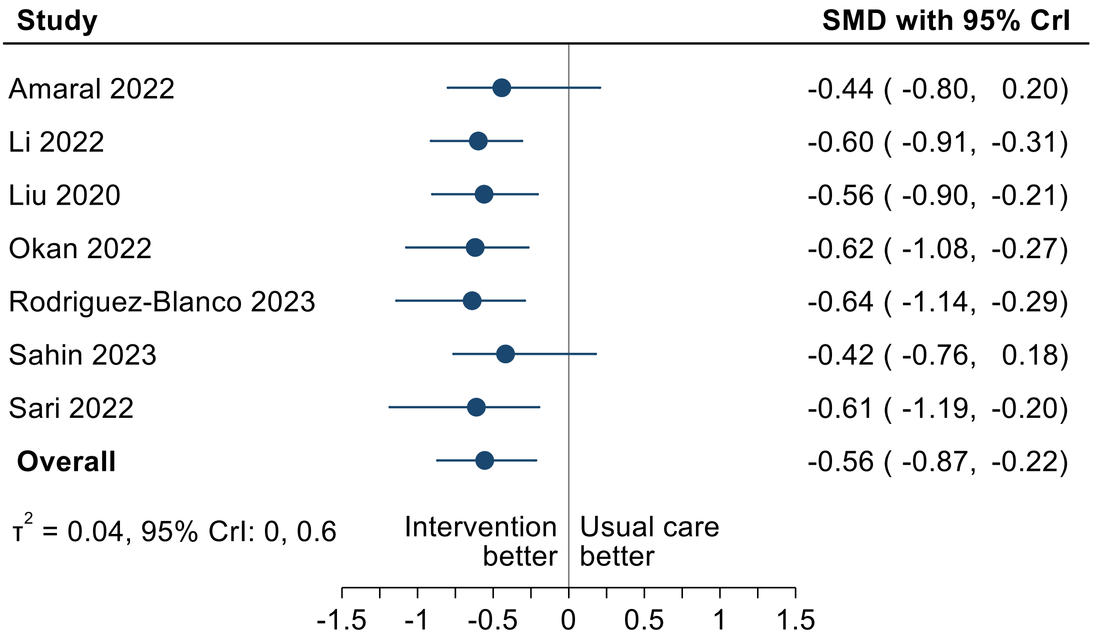

© 2023 Pouliopoulou DV et al. *JAMA Network Open.*

eFigure 2. Caterpillar plot for lower limb muscle function

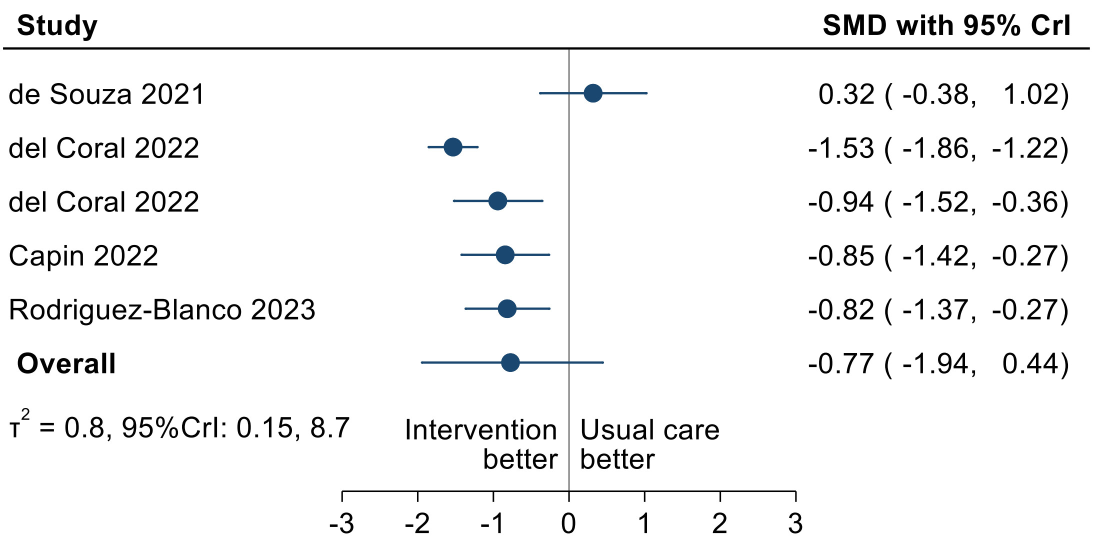

© 2023 Pouliopoulou DV et al. *JAMA Network Open.*

eFigure 3. Caterpillar plot for dyspnea

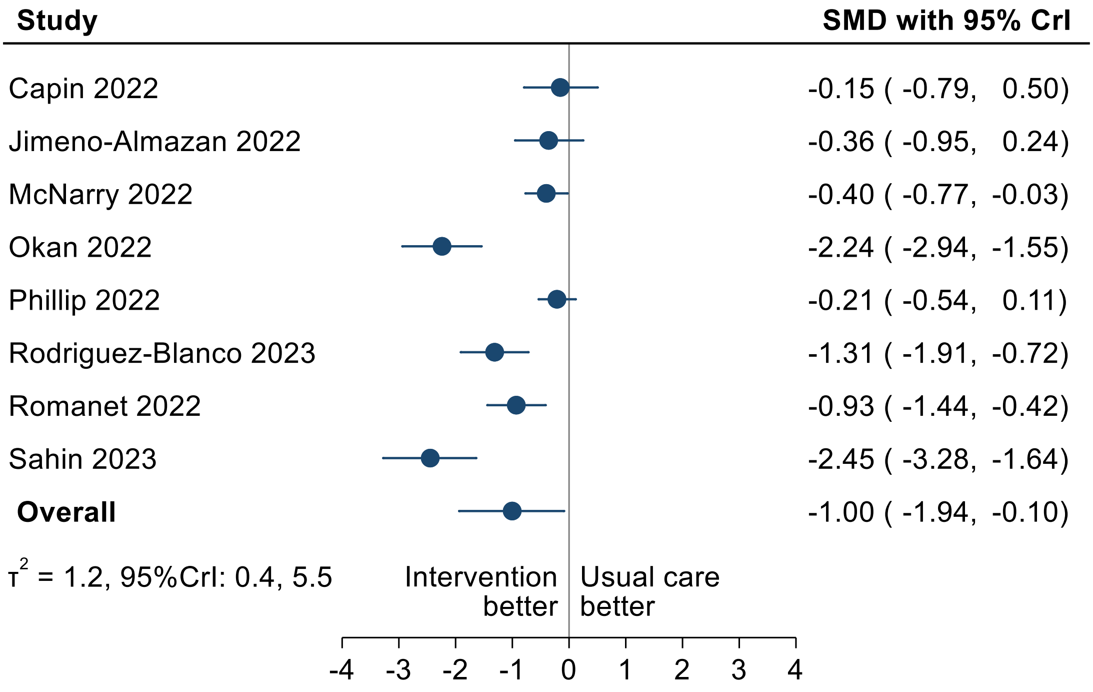

© 2023 Pouliopoulou DV et al. *JAMA Network Open.*

eFigure 4. Sensitivity analysis for dyspnea (allocation concealment)

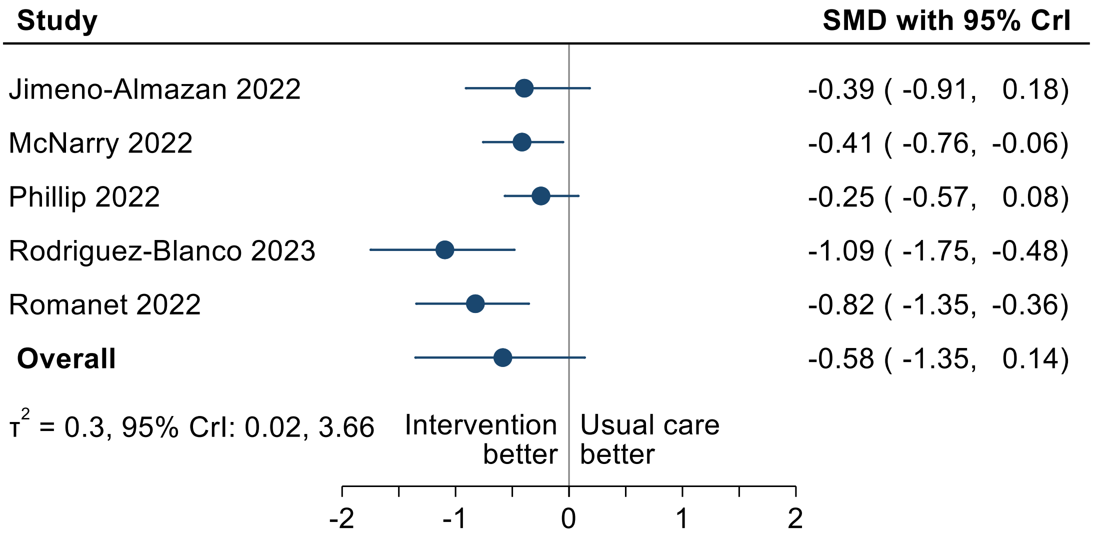

© 2023 Pouliopoulou DV et al. *JAMA Network Open.*

eFigure 5. Caterpillar plot for respiratory function (FEV1)

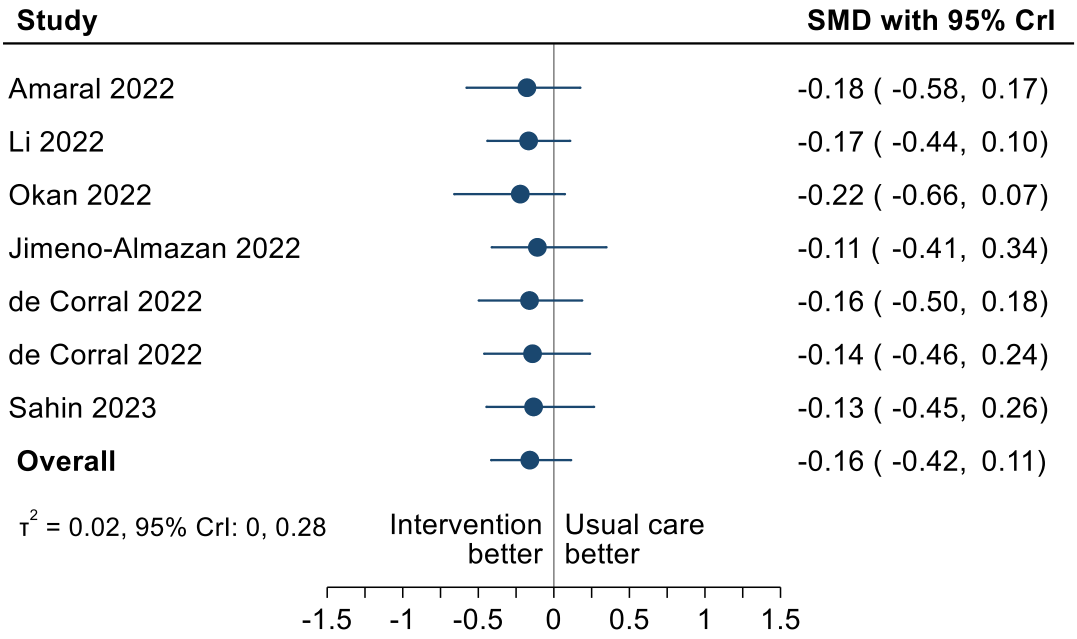

© 2023 Pouliopoulou DV et al. *JAMA Network Open.*

eFigure 6. Caterpillar plot for respiratory function (FVC)

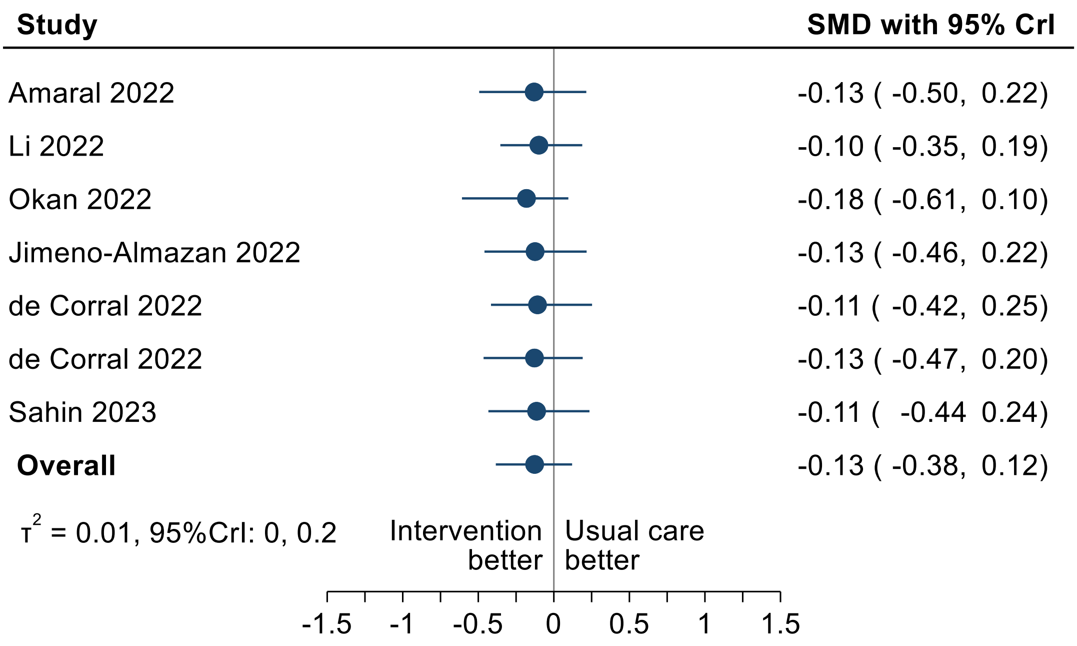

© 2023 Pouliopoulou DV et al. *JAMA Network Open.*

eFigure 7. Caterpillar plot for quality of life outcomes

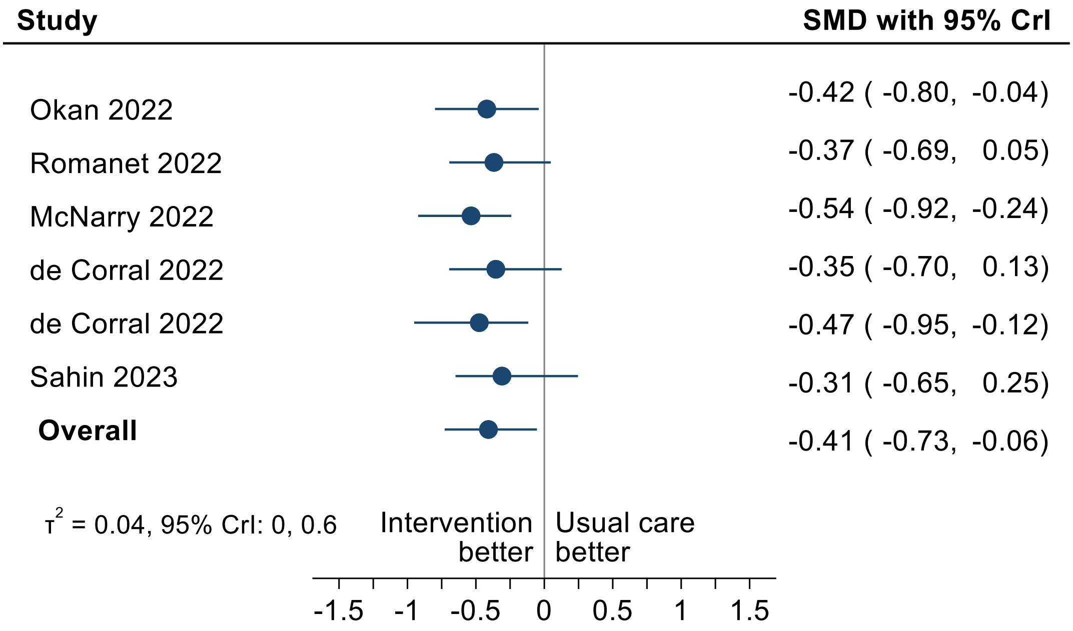

© 2023 Pouliopoulou DV et al. *JAMA Network Open.*

eFigure 8. Caterpillar plot of physical health outcomes

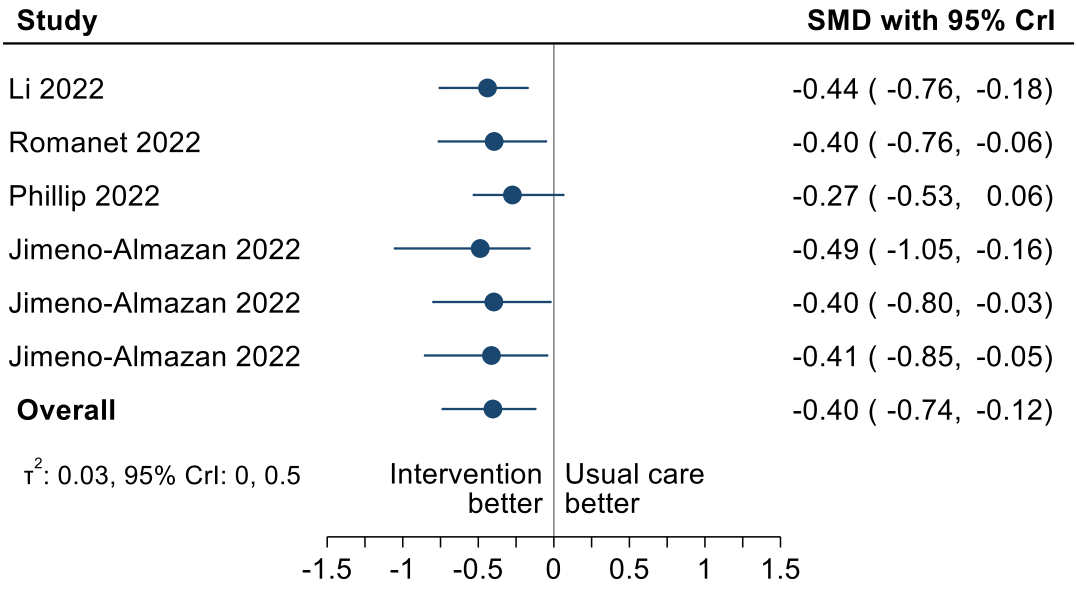

© 2023 Pouliopoulou DV et al. *JAMA Network Open.*

eFigure 9. Caterpillar plot for mental health outcomes

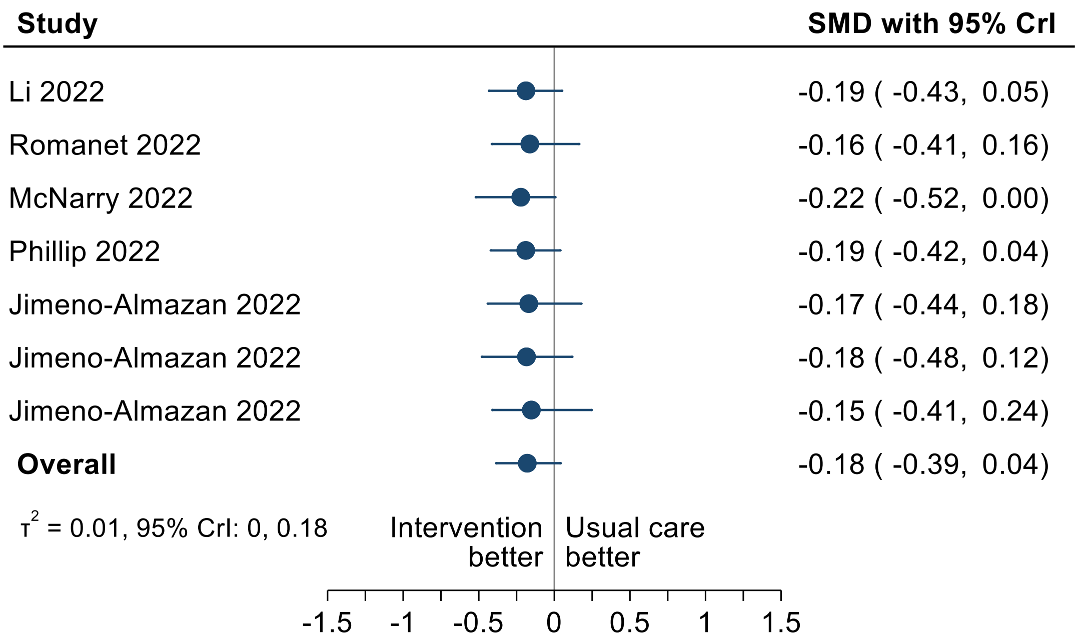

© 2023 Pouliopoulou DV et al. *JAMA Network Open.*

eTable 4. Adverse events Results

	Odds Ratio			Heterogeneity	
**Model**	**Median**	**95% CrI**	**Probability of AEs**	**Median**	**95% CrI**
Fixed Effect	1.45	0.65, 3.40	82%	-	-
Random Effects	1.68	0.32, 9.94	75%	0.30	0.07, 117.70

© 2023 Pouliopoulou DV et al. *JAMA Network Open.*

eTable 5: Model fit and model comparison for adverse events

Summary	Fixed effect model	Random effects model
Posterior mean residual deviances	35.18	25.58
Posterior mean deviances	40.91	33.17
Effective number of parameters	5.73	7.60
Deviance information criteria (DIC)	46.65	40.77

© 2023 Pouliopoulou DV et al. *JAMA Network Open.*

eTable 6: Publication bias

Outcome	SE	P value
Functional aerobic capacity	1.93	0.71
Functional lower limb muscle strength and endurance	2.09	0.25
Dyspnoea	5.01	0.13
Respiratory Function – FEV1	2.54	0.97
Respiratory Function – FVC	2.85	0.88
Quality of life – Overall	3.87	0.60
Quality of life – Physical Health	2.50	0.66
Quality of life – Mental Health	2.44	0.87
Adverse Events	52.26	0.07

© 2023 Pouliopoulou DV et al. *JAMA Network Open.*

eFigure 10. Autocorrelation

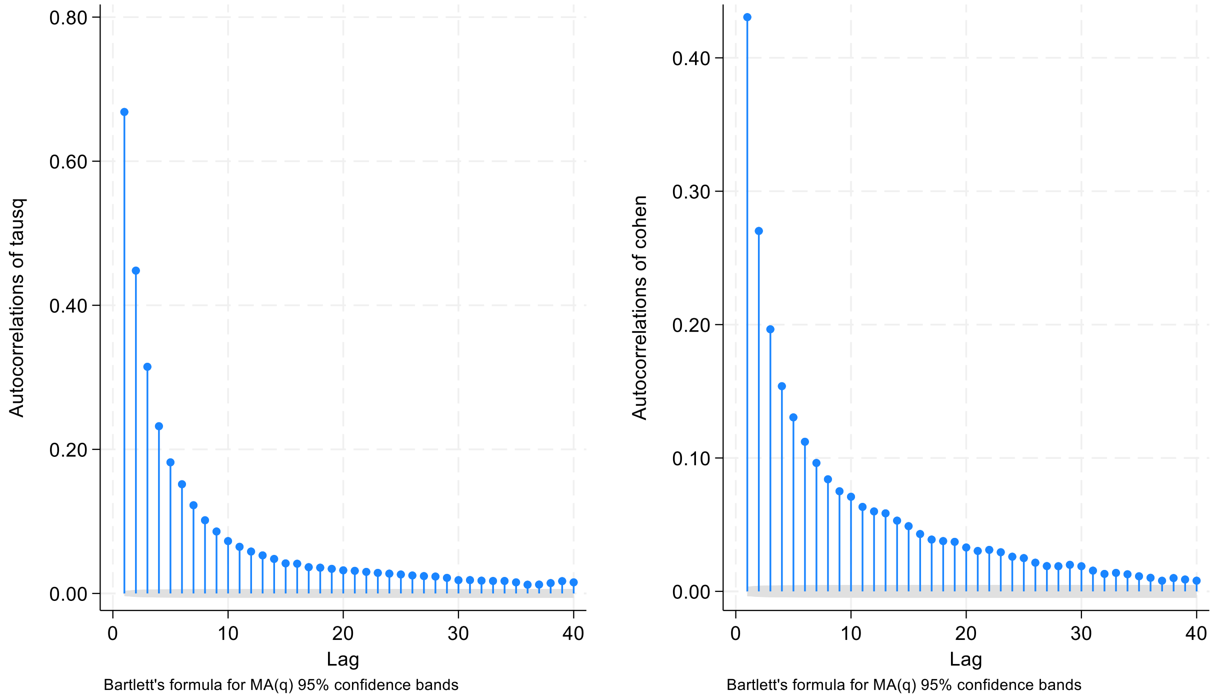

© 2023 Pouliopoulou DV et al. *JAMA Network Open.*

eFigure 11. Trace plot

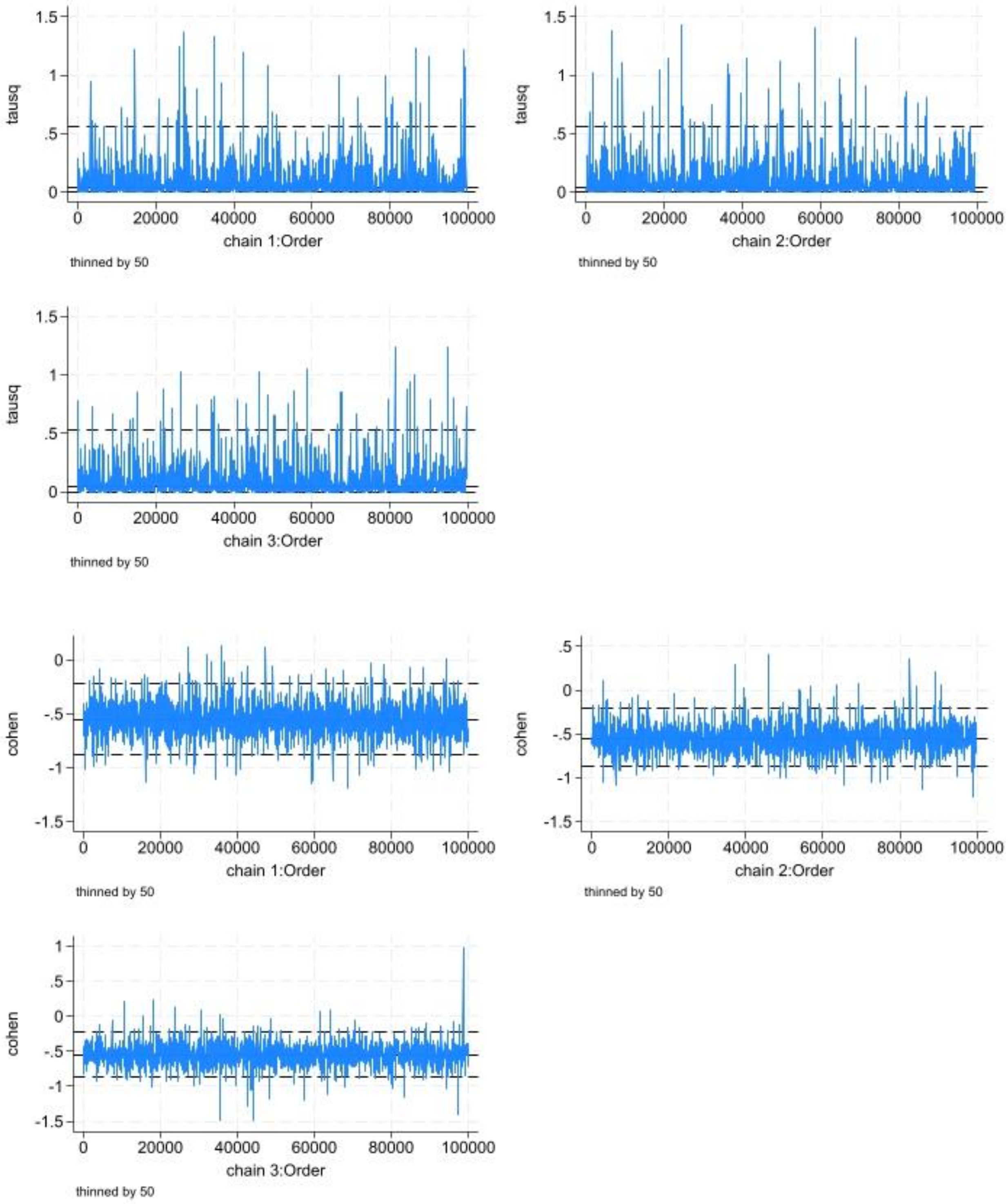

© 2023 Pouliopoulou DV et al. *JAMA Network Open.*

eTable 7. Sensitivity analysis

6MWT				
**Study**	**median**	**95% CrI**	**Pr_Sup**	**Pr_MID**
Amaral 2022	-0.51	(-0.80, -0.01)		
Li 2022	-0.59	(-0.86, -0.33)		
Liu 2020	-0.56	(-0.85, -0.27)		
Okan 2022	-0.59	(-0.96, -0.31)		
Rodriguez-Blanco 2023	-0.60	(-1.00, -0.32)		
Sahin 2023	-0.49	(-0.77, -0.00)		
Sari 2022	-0.59	(-1.01, -0.26)		
**Total effect estimate**	**-0.56**	**(-0.81, -0.31)**	**100.0%**	**97.7%**
**τ^2**	0.01	(0.00, 0.20)		

STS				
**Study**	**median**	**95% CrI**	**Pr_Sup**	**Pr_MID**
de Souza 2021	0.18	(-0.54, 0.90)		
del Coral 2022	-1.51	(-1.83, -1.18)		
del Coral 2022	-0.93	(-1.49, -0.37)		
Capin 2022	-0.85	(-1.39, -0.29)		
Rodriguez-Blanco 2023	-0.83	(-1.35, -0.28)		
**Total effect estimate**	**-0.79**	**(-1.50, -0.02)**	**98%**	**92%**
**τ^2**	-0.92	(-2.50, 0.86)		

Dyspnoea				
**Study**	**median**	**95% CrI**	**Pr_Sup**	**Pr_MID**
Capin 2022	-0.19	(-0.82, 0.45)		
Jimeno-Almazan 2022	-0.38	(-0.97, 0.21)		
McNarry 2022	-0.41	(-0.77, -0.05)		
Okan 2022	-2.18	(-2.88, -1.50)		
Phillip 2022	-0.22	(-0.54, 0.10)		
Rodriguez-Blanco 2023	-1.30	(-1.89, -0.72)		
Romanet 2022	-0.93	(-1.44, -0.42)		
Sahin 2023	-2.36	(-3.18, -1.56)		
**Total effect estimate**	**-0.99**	**(-1.75, -0.28)**	**99%**	**97%**
**τ^2**	0.79	(0.26, 2.92)		

FEV1				
**Study**	**median**	**95% CrI**	**Pr_Sup**	**Pr_MID**
Amaral 2022	-0.17	(-0.49, 0.13)		
Li 2022	-0.16	(-0.42, 0.09)		
Okan 2022	-0.19	(-0.55, 0.07)		
Jimeno-Almazan 2022	-0.13	(-0.40, 0.23)		
de Corral 2022	-0.16	(-0.45, 0.14)		
de Corral 2022	-0.15	(-0.42, 0.16)		
Sahin 2023	-0.14	(-0.41, 0.18)		
**Total effect estimate**	-0.16	(-0.39, 0.08)	**91%**	**11%**
**τ^2**	**0.01**	**(0.00, 0.11)**		

FVC				
**Study**	**median**	**95% CrI**	**Pr_Sup**	**Pr_MID**
Amaral 2022	-0.13	(-0.43, 0.16)		
Li 2022	-0.11	(-0.35, 0.15)		
Okan 2022	-0.16	(-0.50, 0.10)		
Jimeno-Almazan 2022	-0.12	(-1.39, -0.29)		
de Corral 2022	-0.11	(-0.39, 0.19)		

© 2023 Pouliopoulou DV et al. *JAMA Network Open.*

de Corral 2022	-0.13	(-0.42, 0.15)		
Sahin 2023	-0.12	(-0.40, 0.17)		
**Total effect estimate**	**-0.13**	**(-0.36, 0.10)**	**86%**	**7%**
**τ^2**	0.01	(0.00, 0.10)		

**QoL**				
**Study**	**median**	**95% CrI**	**Pr_Sup**	**Pr_MID**
Okan 2022	-0.42	(-0.73, -0.10)		
Romanet 2022	-0.39	(-0.67, -0.04)		
McNarry 2022	-0.49	(-0.83, -0.23)		
de Corral 2022	-0.39	(-0.68, -0.00)		
de Corral 2022	-0.45	(-0.82, -0.15)		
Sahin 2023	-0.36	(-0.65, 0.08)		
**Total effect estimate**	**-0.41**	**(-0.66, -0.15)**	**100%**	**82%**
**τ^2**	0.01	(0.00, 0.19)		

**PHC**				
**Study**	**median**	**95% CrI**	**Pr_Sup**	**Pr_MID**
Li 2022	-0.42	(-0.71, -0.18)		
Romanet 2022	-0.39	(-0.70, -0.11)		
Phillip 2022	-0.32	(-0.55, -0.01)		
Jimeno-Almazan 2022	-0.44	(-0.89, -0.17)		
Jimeno-Almazan 2022	-0.39	(-0.72, -0.09)		
Jimeno-Almazan 2022	-0.40	(-0.76, -0.10)		
**Total effect estimate**	**-0.40**	**(-0.65, -0.17)**	**100%**	**81%**
**τ^2**	0.01	(-2.50, 0.86)		

**MHC**				
**Study**	**median**	**95% CrI**	**Pr_Sup**	**Pr_MID**
Li 2022	-0.19	(-0.41, 0.03)		
Romanet 2022	-0.17	(-0.40, 0.10)		
McNarry 2022	-0.21	(-0.47, 0.00)		
Phillip 2022	-0.19	(-0.40, 0.02)		
Jimeno-Almazan 2022	-0.17	(-0.41, 0.11)		
Jimeno-Almazan 2022	-0.18	(-0.44, 0.08)		
Jimeno-Almazan 2022	-0.16	(-0.39, 0.15)		
**Total effect estimate**	-0.18	(-0.37, 0.02)	**97%**	**11%**
**τ^2**	**0.01**	**(0.00, 0.08)**		

© 2023 Pouliopoulou DV et al. *JAMA Network Open.*

eFigure 12 Sensitivity analysis – Autocorrelation plot

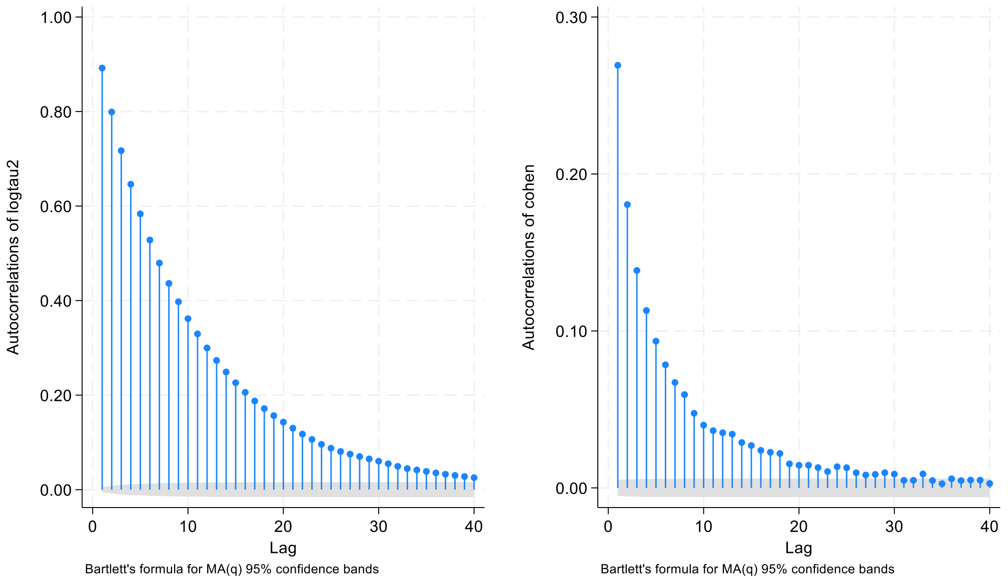

© 2023 Pouliopoulou DV et al. *JAMA Network Open.*

eFigure 13 Sensitivity analysis – Trace plot

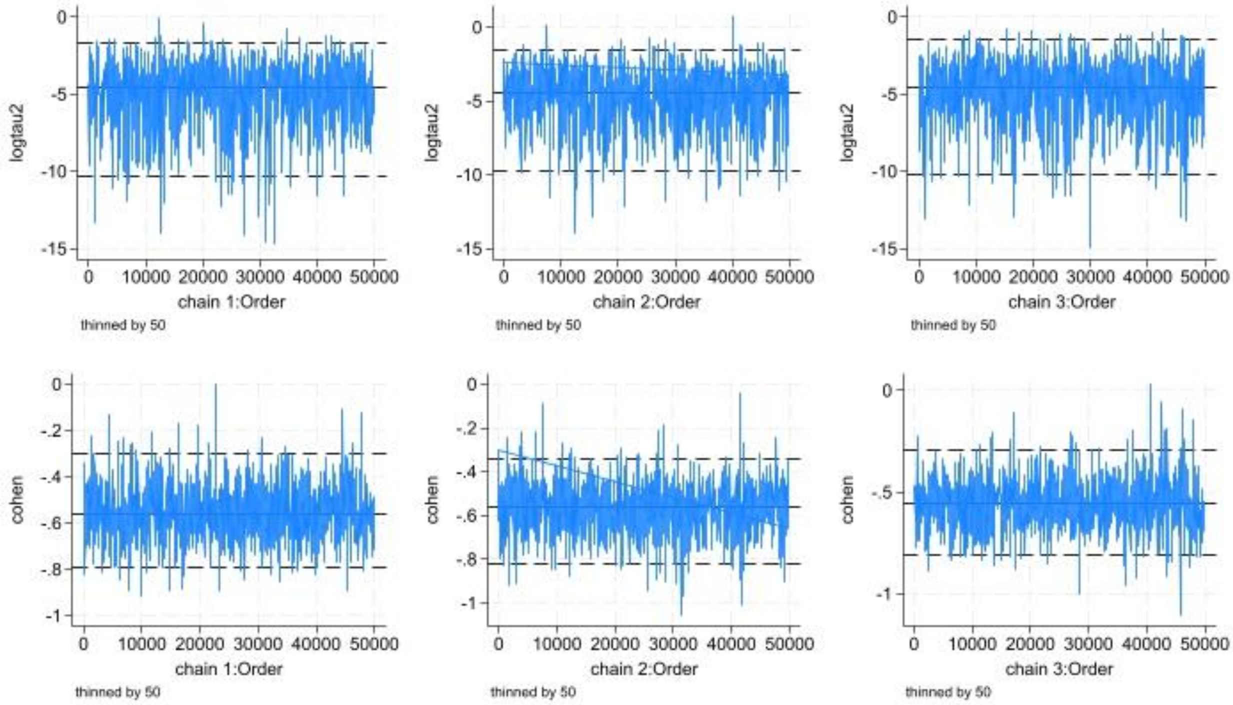

© 2023 Pouliopoulou DV et al. *JAMA Network Open.*
