# Supplementary material for: Rehabilitation Interventions for Physical Capacity and Quality of Life in Adults With Post–COVID-19 Condition: A Systematic Review and Meta-Analysis
Source: JAMA Netw Open. 2023 Sep 19;6(9):e2333838. doi: 10.1001/jamanetworkopen.2023.33838 (PMC10509723; doi:10.1001/jamanetworkopen.2023.33838)
Supplement: Supplement 2. — Data Sharing Statement [file jamanetwopen-e2333838-s002.pdf]

Data Sharing Statement

Pouliopoulou. Rehabilitation Interventions for Physical Capacity and Quality of Life in Adults With Post–COVID-19 Condition. *JAMA Netw Open*. Published September 19, 2023. doi:10.1001/jamanetworkopen.2023.33838

Data
Data available: Yes
Data types: Data (not involving human participants)
How to access data: The guarantor (DVP) is willing to examine all requests for the full dataset after two years from the date of this publication.
When available: beginning date: 06-01-2025

Supporting Documents
Document types: None

Additional Information
Who can access the data: researchers whose proposed use of the data has been approved
Types of analyses: for any purpose
Mechanisms of data availability: after approval of a proposal
